# Disrupting pro-survival and inflammatory pathways with dimethyl fumarate sensitizes chronic lymphocytic leukemia to cell death

**DOI:** 10.1038/s41419-024-06602-z

**Published:** 2024-03-18

**Authors:** Maria Elena Mantione, Miriam Meloni, Ilenia Sana, Jessica Bordini, Martina Del Nero, Michela Riba, Pamela Ranghetti, Eleonora Perotta, Paolo Ghia, Lydia Scarfò, Marta Muzio

**Affiliations:** 1 Cell Signaling Unit, Division of Experimental Oncology, IRCCS Ospedale San Raffaele, Milano, Italy; 2 B-cell Neoplasia Unit, Division of Experimental Oncology, IRCCS Ospedale San Raffaele, Milano, Italy; 3 Center for Omics Sciences, IRCCS Ospedale San Raffaele, Milano, Italy; 4 Università Vita-Salute San Raffaele, Milano, Italy

**Keywords:** Preclinical research, Chronic lymphocytic leukaemia

## Abstract

Microenvironmental signals strongly influence chronic lymphocytic leukemia (CLL) cells through the activation of distinct membrane receptors, such as B-cell receptors, and inflammatory receptors, such as Toll-like receptors (TLRs). Inflammatory pathways downstream of these receptors lead to NF-κB activation, thus protecting leukemic cells from apoptosis. Dimethyl fumarate (DMF) is an anti-inflammatory and immunoregulatory drug used to treat patients with multiple sclerosis and psoriasis in which it blocks aberrant NF-κB pathways and impacts the NRF2 antioxidant circuit. Our in vitro analysis demonstrated that increasing concentrations of DMF reduce ATP levels and lead to the apoptosis of CLL cells, including cell lines, splenocytes from Eµ-TCL1-transgenic mice, and primary leukemic cells isolated from the peripheral blood of patients. DMF showed a synergistic effect in association with BTK inhibitors in CLL cells. DMF reduced glutathione levels and activated the NRF2 pathway; gene expression analysis suggested that DMF downregulated pathways related to NFKB and inflammation. In primary leukemic cells, DMF disrupted the TLR signaling pathways induced by CpG by reducing the mRNA expression of NFKBIZ, IL6, IL10 and TNFα. Our data suggest that DMF targets a vulnerability of CLL cells linked to their inflammatory pathways, without impacting healthy donor peripheral blood mononuclear cells.

## Introduction

Chronic lymphocytic leukemia (CLL) is a B-cell neoplasia caused by the accumulation of monoclonal malignant mature B lymphocytes in the peripheral blood (PB), bone marrow (BM) and secondary lymphoid tissues [1]. CLL is a prototypic microenvironment-dependent tumor, as, in addition to distinct genomic aberrations, several signaling pathways triggered by transmembrane receptors contribute to the pathobiology of the disease [2–4]. CLL cells maintain some biological and molecular characteristics of the putative B-cell of origin, including the expression of a unique clonotypic B-cell receptor as well as costimulatory receptors that play a fundamental role in their proliferation, accumulation, and survival [5]. Accordingly, several kinase inhibitors block BCR signaling, among others, and are now approved for therapeutic purposes; in particular, BTK inhibitors have been developed and improved to the second and third generation [6–8]. In addition, PI3K inhibitors as well as proapoptotic agents such as BCL2 inhibitors have been approved [9, 10]. Owing to all these therapeutic options, CLL patients can achieve long-lasting remission; however, deep responses (including complete remission and undetectable measurable residual disease) are rarely observed, with all patients eventually relapsing. Thus, novel approaches with different targets are urgently needed, especially for patients who relapse after treatment with both BTK and BCL2 inhibitors.

Among the signaling pathways that have been recently studied and targeted in preclinical models, a prominent role is attributed to the inflammatory cascade that may directly impact tumor cells by increasing apoptosis resistance and chemoresistance in vitro and by inducing cell migration and proliferation in lymphoid tissues [11–14]. Mutations affecting the NF-kB pathway have been observed in a portion of CLL cases [2, 15]. Nonetheless, constitutive activation of NF-kB has been observed in most CLL samples [16, 17], and hyperactivation can be induced by inflammatory receptors, including Toll-like receptors (TLRs), which can be recapitulated in vitro by treating CLL cells with the TLR9 ligand CpG, either alone or in combination with a CD40 ligand and/or IL2 [11, 17–21]. Notably, TLR9 stimulation induces greater levels of the atypical NF-kB family member IkBz in leukemic cells than in B cells isolated from healthy donors, and knocking down the IkBz protein reduced TLR-mediated activation, confirming the functional role of IkBz in this process [22]. Moreover, B-cell-specific TNF receptor family members strongly impact NF-kB activation both in vitro and in vivo [23–25]. Similarly, additional cytokine signaling pathways, including STAT activation, have been implicated in the mechanisms regulating CLL cell viability and proliferation [26, 27]. Overall, these observations suggest that genetic abnormalities as well as microenvironmental stimulation through different surface receptors result in an intrinsic inflammatory state in leukemic cells that may be targeted for therapeutic purposes. In addition, CLL cells show elevated levels of the transcription factor NF-E2-related factor 2 (NRF2), also known as nuclear factor (erythroid-derived 2)-like 2, compared to mononuclear cells isolated from the PB of healthy donors, and several electrophilic compounds exert diverse levels of toxicity against leukemic cells; among them, it was suggested that dimethyl fumarate (DMF) may reduce the viability of CLL cells by electrophilic stress [28].

DMF is a generic drug currently used to treat patients with multiple sclerosis or psoriasis [29–31]. Compared with classic immunosuppressants, it is an anti-inflammatory and immunoregulatory pleiotropic agent with several unique properties. DMF is a prototypic NRF2 activator that acts as an antioxidant in both immune and neuronal cells [32, 33]. DMF strongly reacts with GSH as well as with other proteins containing reactive cysteines, resulting in the succination of several targets. Moreover, it is a well-known inhibitor of the inflammatory NF-kB pathway and the STAT/JAK pathway [29–31]. Notably, it was recently shown that itaconate derivatives and DMF directly impact IkBz expression, blocking the inflammatory cascade induced by TLR4 in mouse macrophages [34, 35]; moreover, DMF could inhibit the interaction and activation of the MyD88-IRAK4 signaling complex that mediates TLR signaling in plasmacytoid dendritic cells [36].

Based on all these observations, we hypothesized that DMF could block inflammatory pathways intrinsic to CLL cells or that are activated after TLR9 triggering and could target the vulnerability of CLL cells linked to both IkBz and NRF2 overexpression. To address this hypothesis, we performed preclinical testing and molecular analysis of DMF*-treated* CLL cell lines, primary CLL cells, and murine leukemic splenocytes in vitro.

## Results

### DMF reduces the growth and metabolic activation of CLL cell lines and induces apoptosis

**Fig. 1:**
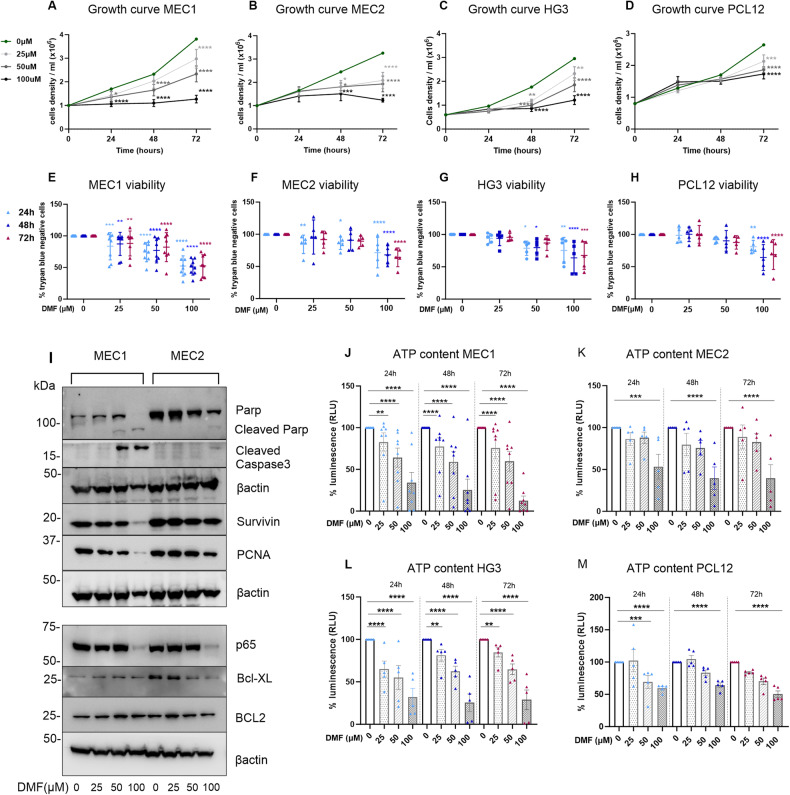
DMF inhibits CLL cell lines proliferation and induces cell death. **A–D** Growth curves of the indicated cell lines treated with increasing concentrations of DMF for 24 h, 48 h and 72 h, expressed as cell number per milliliter. **E**–**H** Cell viability was assessed with trypan blue exclusion method and automatic counting before and after 24 h, 48 h and 72 h of treatment with DMF; data are expressed as percentage of viable cells compared to that of untreated cells. *n* = 8 biological replicates for MEC1; *n* = 5 for the other cell lines for panels A-H. **I** Western blot analysis of proapoptotic and antiapoptotic proteins expression after DMF treatment (25, 50 and 100 µM); images representative of two independent experiments performed with MEC1 and MEC2 total cellular extracts. Beta-actin was used as internal control. **J**–**M** Metabolic activation in CLL cell lines exposed to increasing doses of DMF (25–100 µM) after 24 h, 48 h and 72 h; ATP content is expressed as percentage of relative luminescence units, RLU, versus untreated (MEC1, *n* = 8; MEC2, *n* = 5; HG3, *n* = 5; PCL12, *n* = 5). All the data are shown as the mean ± SD. Two-way ANOVA with Dunnett’s post hoc test was performed for multiple comparisons. *p < 0.05; **p < 0.01; ***p < 0.001; ****p < 0.0001.

First, we measured the capacity of DMF to impact the proliferation of MEC1, MEC2, PCL12 and HG3 CLL cell lines in vitro, and we observed that the total number of cells decreased in a time- and dose-dependent manner (Fig. 1A–D). DMF (100 μM) inhibited proliferation at 48 h in all the cell lines except PCL12. Western blot analysis confirmed a block in cell proliferation, as demonstrated by decreased levels of the proliferating cell nuclear antigen PCNA (Fig. 1I).

Next, to determine whether DMF impacted cell viability, we counted trypan blue-negative cells in treated vs untreated cells at different time points. The relative number of viable cells indicated that DMF induced cell death in a dose-dependent manner (Fig. 1E–H); again, PCL12 cells were the most resistant. Western blot analysis confirmed the dose-dependent induction of apoptosis in MEC1 and MEC2 cells, as demonstrated by increased PARP and Caspase-3 specific cleavage (Fig. 1I). Moreover, DMF partially impacted the total levels of the antiapoptotic protein Survivin and decreased the levels of BCL-XL but not of BCL2, especially in the MEC2 cell line (Fig. 1I**)**. Notably, the NFkB transcription factor p65 was significantly reduced by DMF in both MEC1 and MEC2 cells (Fig. 1I and Supplementary Figure 1).

The relative number of viable cells can also be calculated based on the quantitation of the ATP present in the cell culture, an indicator of metabolically active cells. Using the Cell Titer Glo (CTG) assay, we detected marked ATP impairment in all the cell lines after DMF treatment in a dose-dependent manner starting from 25 to 100 μM from 24 to 72 h. Again, MEC2 and PCL12 were more resistant (Fig. 1J–M). Overall, these analyses demonstrated that DMF diminished ATP content and induced cell death in all the cell lines, although the effects on MEC2 and PCL12 cells were less pronounced than those on MEC1 and HG3 cells.

We calculated the half maximal inhibitory concentration (IC50) at 48 h for all the cell lines analyzed and confirmed that DMF is a potent inhibitor of cell line viability, with IC50 values ranging from 37 to 124 (37.25 μM for MEC1, 76.37 μM for MEC2, 69.55 μM for HG3 and 124.1 μM for PCL12). These concentrations are in line with the active concentrations of DMF previously reported for immunoregulatory activity in vitro [35, 37–40].

### DMF reduces primary leukemic cell survival without impairing healthy leukocytes

**Fig. 2:**
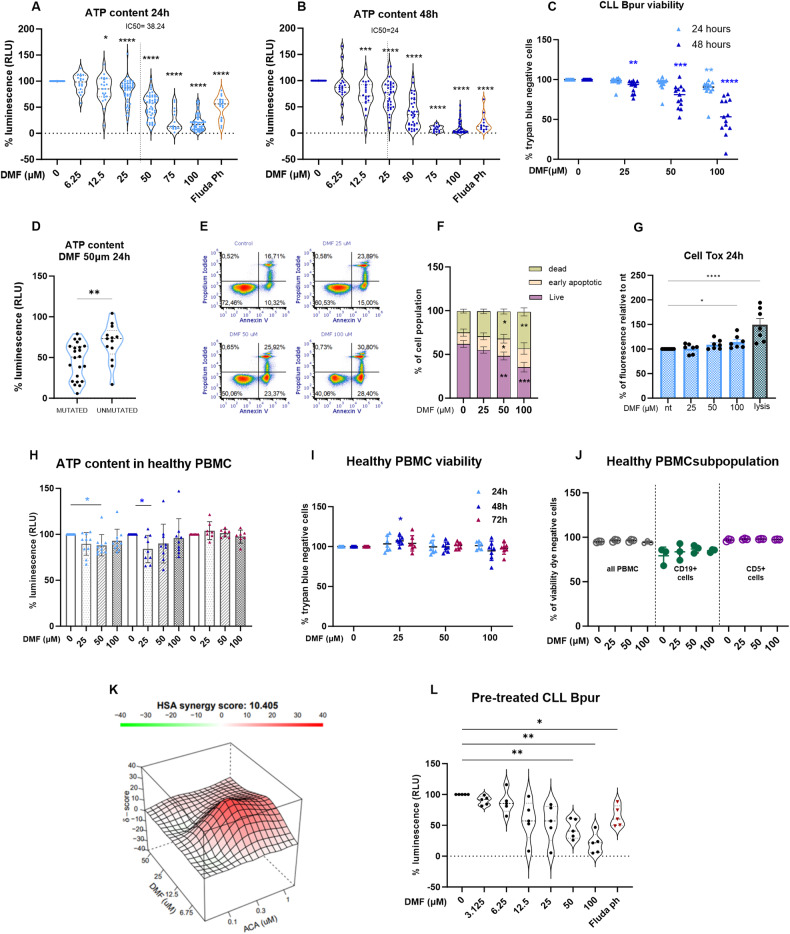
DMF reduces the viability and metabolic activation of primary CLL cells. Increasing concentrations of DMF from 6.25 to 100 µM were applied to CLL cells for 24 h (**A** *n* = 51) and 48 h (**B** *n* = 35); following, cell titer assay was performed, and data are expressed as the percentage of metabolic activation compared to untreated. Two-way ANOVA was performed, followed by Dunnett’s post hoc test for multiple comparisons. *p < 0.05; **p < 0.01; ***p < 0.001, ****p < 0.0001. The calculated IC50 values at 24 h (**A**) and 48 h (**B**) are reported. **C** Viability of CLL patients’ leukemic cells at 24 h and 48 h after in vitro culture with or without DMF (25, 50 and 100 µM); cell viability was assessed with the trypan blue exclusion method and automatic counting, and the results are shown as the percentage of viable cells compared to that of untreated cells (*n* = 14, mean ± SD). **D** DMF-mediated effects in terms of reduced ATP levels are reported in IGHV-mutated versus IGHV-unmutated CLL samples. **E** Representative plot of Annexin V and propidium iodide (PI) flow cytometry analysis of CLL primary samples treated with DMF. **F** Bar graph showing the percentage of alive, early apoptotic, and dead CLL cells after 24 h of DMF treatment (*n* = 8). The means ± SEMs are shown, and one-way ANOVA was applied. **G** Relative toxicity was evaluated by Cell Tox Green in CLL cells treated with DMF for 24 h. Evaluation of the ATP content (**H**) and relative cell viability (**I**) of PBMCs isolated from healthy donors at 24 h (*n* = 10), 48 h (*n* = 9) and 72 h (*n* = 7) after treatment with DMF at 25, 50, or 100 µM (mean ± SD). **J** Flow cytometry analysis of cell viability in PBMCs or B- or T-cell compartments as assessed by staining with CD5, CD19 and viability dye; PBMCs were exposed to DMF for 24 h before analysis (*n* = 3). **K** CLL cells were treated in vitro with increasing concentrations of DMF plus acalabrutinib (*n* = 8). Metabolic activation was measured by cell titer, and relative ATP values were used to calculate the combination index. **L** CLL cells were isolated from the peripheral blood of patients undergoing treatment with a BTK inhibitor; the percentage of relative ATP content in samples treated in vitro for 24 h with the indicated concentrations of DMF is reported. The Friedman test was performed (*n* = 5). *p < 0.05, **p < 0.01, ***p < 0.001.

Prompted by the encouraging results obtained from cell lines, we investigated the in vitro effects of increasing concentrations of DMF on primary CLL cells. We measured the ATP content and observed a significant reduction after both 24 and 48 h with DMF, which was already active at 12.5 µM (Fig. 2A, B). Fludarabine phosphate was included as an internal control (Fluda Ph). We calculated that the IC50 of DMF was 38.24 μM at 24 h and 24 μM at 48 h (*n* = 51 at 24 h, *n* = 35 at 48 h). Because the main metabolite of DMF in vivo is monomethyl fumarate (MMF) [29–31], which was previously shown to be less active than DMF in different cellular assays [35–37, 41], we analyzed, as proof of principle, 5 cases: increasing concentrations of MMF had a significant impact on cell viability, although the effect was lower than that of DMF (Supplementary Fig. 2D, E). Notably, at a concentration of 100 μM, DMF reduced cell viability in all the samples analyzed independently of the mutational status of the IGHV genes**;** however, at 50 μM, DMF had a greater effect on the mutated samples (Fig. 2D and Supplementary Fig. 2A). These results were paralleled at lower levels by an increase in cell death, as assessed by Trypan blue dye staining (Fig. 2C); the induction of apoptosis was evident after Annexin-PI staining (Fig. 2E for representative sample), which revealed a significant increase in both early and late apoptosis (Fig. 2F). Cytotoxicity was assessed by a specific assay (Cell Tox Green) in 7 samples, and a mild but significant effect was confirmed.

We performed additional experiments by adding a feeder layer (human HS5 cell line), and we observed a significant decrease in the ATP content in DMF-treated cells isolated from 4 patients even in the presence of the cytoprotective effects of stroma. DMF was not toxic to stromal cells under the same experimental conditions (Supplementary Fig. 2B, C).

To evaluate whether the toxicity of DMF also occurred in normal leukocytes under these experimental conditions, we treated PBMCs isolated from healthy donors following the same protocol described above, and we observed that DMF did not affect the viability of normal PBMCs even at the highest dose of DMF (100 μM) at any of the time points analyzed (Fig. 2H–J). The ATP content slightly decreased with low doses of DMF at 24–48 h but returned to the initial levels at 72 h after treatment (Fig. 2H). Moreover, to determine whether DMF had a specific antileukemic effect or targeted the B-cell lineage, we treated healthy PBMCs with 100 µM DMF and analyzed cell viability after 24 h by flow cytometry staining with anti-CD5 and anti-CD19 antibodies (marking T cells or B cells, respectively); no significant difference in viable B/T-cell distribution was observed (Fig. 2J). These results show that DMF selectively inhibited leukemic cells without affecting normal B cells.

### DMF synergizes with acalabrutinib in vitro

To assess whether DMF interacted with currently used drugs, we combined DMF with the BTK inhibitor acalabrutinib or with the BCL2 inhibitor venetoclax. We also included a BCL2 family inhibitor (Navitoclax) in the analysis. After 24 and 48 h of incubation in vitro, we measured ATP content in a small cohort of naive patient samples. As expected, acalabrutinib alone led to only partial cell death in vitro [42]; analysis of the cotreatment results at 48 h showed global synergism between DMF and acalabrutinib (*n* = 8), with a score of 10.405 calculated with the highest single agent (HSA) reference model [43, 44]; the most synergistic score area was between 6.75 µM and 25 µM for DMF and between 0.3 and 3 µM for acalabrutinib (synergy score = 17.03; Fig. 2K). Nevertheless, the combination score of DMF with acalabrutinib was quite heterogeneous among different cases: 7 patients exhibited synergistic or additive effect, while one cases displayed only an antagonistic effect. In contrast, the calculated combination index of DMF and venetoclax (*n* = 6) or navitoclax (*n* = 5) showed no synergistic effect (Supplementary Fig. 2F, G).

Next, we tested the cytotoxic activity of DMF on CLL cells obtained from 5 additional patients who were under treatment with BTK inhibitors at the time of blood sampling (see Supplementary Table 2 for the clinico-biological characteristics and ongoing treatments): we observed a dose-dependent therapeutic effect even in this population (Fig. 2I).

#### DMF depletes the GSH pool and induces NRF2 activation

**Fig. 3:**
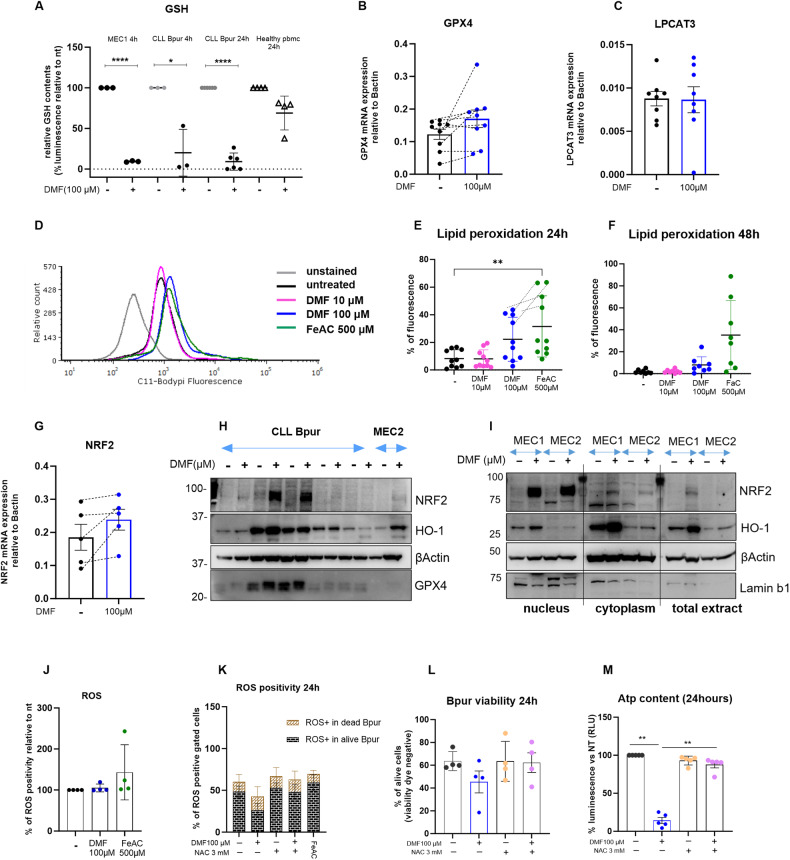
DMF depletes the GSH pool and induces NRF2 activation. **A** Relative level of reduced glutathione (GSH) in purified CLL cells (Bpur) or PBMCs isolated from healthy donors before and after DMF treatment for 4 h (*n* = 3) or 24 h (*n* = 6 for CLL Bpur and *n* = 4 for PBMCs) as indicated. The data are presented as the percentage of relative luminescence units normalized to the untreated. The data are presented as the means ± SDs, and Student’s t test was applied. **B**, **C**, **G** GPX4 (*n* = 9), LPCAT3 (*n* = 8) and NRF2 (*n* = 5) mRNA expression levels relative to those of beta-actin were evaluated by qRT‒PCR in CLL samples treated for 4 h with DMF. The bar graph shows the mean ± SD, and Student’s t test was used to compare two groups. Representative histograms of flow cytometry analysis (**D**) and quantification of lipid peroxidation by flow cytometry using Bodypi C11 at 24 h (*n* = 10, **E**) and 48 h (*n* = 8, **F**) (mean ± SD). **H** Immunoblotting for GPX4, NRF2 and its target HO-1 in whole-cell lysates of CLL samples (*n* = 5) and MEC2 cells with or without 100 µM DMF for 4 h. **I** Immunoblot analysis of NRF2 and HO-1 proteins in the MEC1 and MEC2 cell lines. The expression in nuclear, cytoplasmic or total fractions was analyzed. Bactin and lamin B1 were used as internal controls. **J** Ros production detected by flow cytometry in DMF-treated cells compared to untreated cells (4 h). **K** Ros positivity in dead or alive CLL cells treated for 24 h with DMF alone or in combination with NAC. NAC restored viability (**L**) and ATP levels (**M**) of CLL cells treated with DMF for 24 h.

Changes in the levels of the major cellular antioxidant glutathione (GSH) may lead to different forms of cell death, including apoptosis, ferroptosis and autophagy [45, 46]. Given that DMF was previously reported to succinate GSH in other cellular models, we tested the ability of DMF to modulate the content of GSH in CLL cells. GSH decreased after 4 h of DMF treatment in both MEC1 and primary leukemic cells, where complete depletion occurred at 24 h (Fig. 3A). In contrast, even 24 h of DMF treatment of healthy PBMCs exerted no significant depletion, with only a minor trend of decrease.

Since GSH is the co-factor of GPX4, a key enzyme in catalyzing lipid peroxides, we analyzed GPX4 mRNA expression after 4 h of exposure to DMF, and we detected an increase in the expression of GPX4 in 6 out of 9 samples, although the increase was not statistically significant at the cohort level (Fig. 3B). Heterogeneous levels of GPX4 protein were detected in CLL samples (*n* = 5) but were not significantly changed after treatment with DMF (Fig. 3H and Supplementary Fig. 3D–F).

Notably, DMF was previously described to induce ferroptosis in lymphoma [46]; to determine whether ferroptosis, in addition to apoptosis, occurs, we analyzed lipid peroxidation, a hallmark of this type of cell death. We detected increased lipid peroxidation at 24 h after DMF exposure in 4 patients out of 10 analyzed, notably the same with higher peroxidation after ferric ammonium citrate (FeAc) treatment (Fig. 3D, E); however, after 48 h of DMF treatment, low peroxidation levels were restored (Fig. 3F). Lysophosphatidylcholine acyltransferase 3 (LPCAT3), which promotes ferroptosis by providing a substrate for oxidative damage, did not change at the mRNA level (Fig. 3C).

Previous studies reported DMF-mediated NRF2 activation in multiple sclerosis [47, 48] as a readout of the oxidative stress response. We thus monitored NRF2 levels, and while NRF2 mRNA was only slightly increased after 4 h of DMF treatment, protein expression in total cellular extracts was increased, though variable among CLL cases (Fig. 3G, H). Since NRF2 needs to translocate into the nucleus to activate target genes, we separated nuclear and cytoplasmic fractions from the MEC1 and MEC2 cell lines after 4 h of DMF treatment. Increased nuclear expression of NRF2 in both cell lines compared to untreated cells was detected, as well as an increase of its target gene heme oxygenase 1 (HO-1) (especially in the cytoplasm; Fig. 3I). To confirm that nuclear translocation correlated with NRF2 activation, we functionally tested the binding capacity of NRF2 to NRF2-specific target sequences and found that DMF activated NRF2 in the nucleus 4 h after treatment (MEC1 in Supplementary Fig. 3A). As a proof of principle, we confirmed NRF2 translocation in the nucleus of one patient sample (Supplementary Fig. 3B).

These data indicate that the depletion of GSH (which usually induces oxidative stress) is followed by the activation of NRF2, which is a key antioxidant transcription factor; therefore, we analyzed reactive oxygen species (ROS) production. Surprisingly, flow cytometry analysis detected the same ROS levels in untreated and DMF-treated cells after 4 h, and a trend of decrease after 24 h preceding cell death.

Next, we incubated the cells with N-acetylcysteine (NAC), an antioxidant agent and cell-permeable donor of cysteines that can increase GSH levels. NAC protected CLL cells from DMF-induced cell death and restored ATP content after 24 h (Fig. 3L, M; *n* = 4 and *n* = 5, respectively).

Overall, DMF activity is exerted mainly by ATP and GSH depletion, followed by apoptosis. On the one hand, prolonged NRF2 activation may dampen ROS production, while on the other hand, it may not be sufficient to protect from cell death.

#### DMF shapes a distinct transcriptional program in CLL cells

**Fig. 4:**
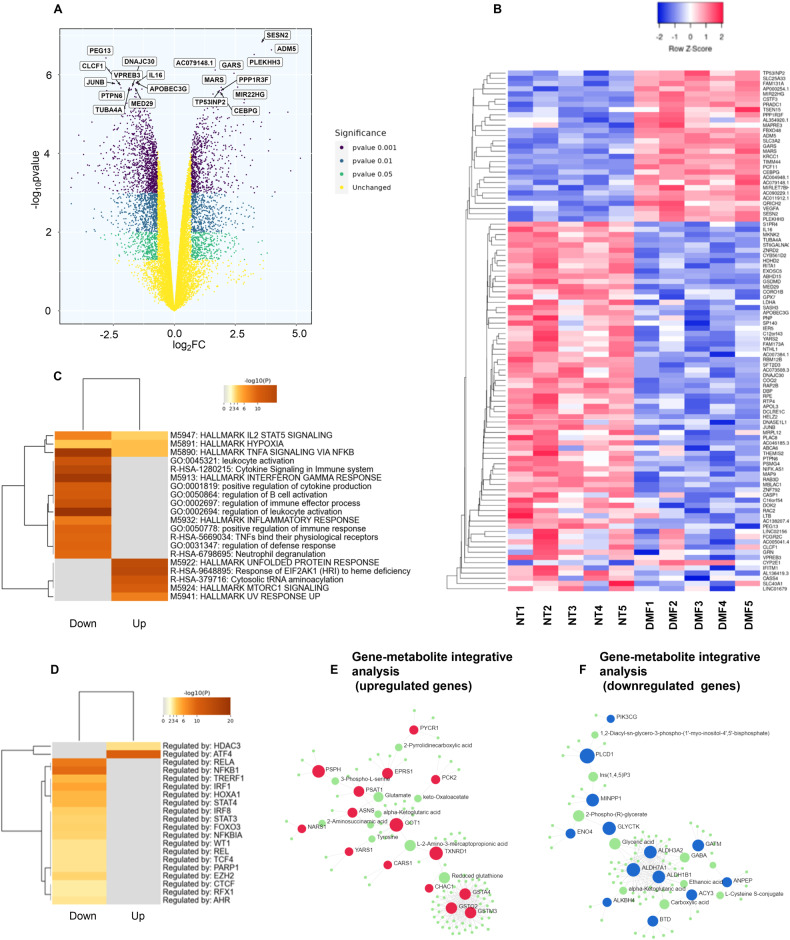
DMF triggers a specific transcriptional program in CLL cells. **A–D** RNA-sequencing data of 5 CLL samples treated with DMF and untreated cells were analyzed. **A** Volcano plot of DMF-treated compared to untreated CLL cells; the X-axis denotes the values of Log_2_FC, and the Y-axis denotes the values of Log_10_pValue. Significant genes are defined by |Log_2_FC| ≥ 1 and different pValues: pValue between ≤ 0.05 and >0.01 (green); pValue between ≤0.01 and >0.001; (blue); pValue ≤ 0.001 (violet). Unchanged genes are represented in yellow. **B** Heatmap showing the clustered expression values of the top 100 DEGs between CLL cells treated with 100 µM DMF for 4 h and untreated. Genes are flagged as “differentially expressed” if they satisfy both the following conditions: nominal p value < 0.01 and LogFC ≥1. A three-color scheme was used, with blue indicating downregulated and red upregulated. Values have been scaled in rows. The color key is displayed at the top of the heatmap. **C** Enrichment map. Enrichment analysis was performed with Metascape, and the top 20 enriched pathways among all the lists are shown. **D** Specific analysis of the transcription factor TRRUST was performed with Metascape, and the top 20 enriched factors (downregulated or upregulated) are shown. **E**, **F** Gene‒metabolite integrative analysis performed with Omics.net. Metabolites are represented in green, and genes are represented in red for upregulated and in blue for downregulated DEGs.

To characterize the genes involved in DMF-mediated effects, we performed RNA sequencing of primary leukemic cells that were either untreated or treated in vitro with 100 µM DMF for 4 h. From the analysis of 5 CLL patient samples, we identified 633 upregulated genes and 1013 downregulated genes. (Fig. 4A). Among these genes, PEG13, VPREB3, IL16, APOBEC3G, and JUNB were the 5 most downregulated, whereas SESN2, ADM5, PLEKHH3, GARS, and MIR22HG were the 5 most upregulated (Fig. 4B).

All DEGs downregulated and upregulated by DMF were further analyzed using Metascape to profile the global interference of biological pathways [49]. Among the most enriched pathways by analyzing downregulated genes, several immune-related signatures and inflammatory pathways emerged, further confirming the anti-inflammatory action of DMF. The analysis of upregulated genes highlighted stress response pathways, the UPR, cytosolic tRNA aminoacylation and mTORC1 (Fig. 4C). Enrichment analysis against transcriptional regulatory network connection (TRRUST) showed upregulation of HDAC3 and ATF4 transcription factor profiles in DMF-treated cells, while the NFkB family together with STAT3 and EZH2 emerged among the downregulated transcription factors (Fig. 4D), further reinforcing the results from the gene expression analysis.

Then, we performed an integrative analysis of protein–metabolism molecular interactions with the OmicsNET tool. From analysis of upregulated DEGs emerged an enrichment in glutathione metabolism (n.Hits = 11, adj.p value = 8.24e-08), represented by interaction between the glutathione S-Transferase enzymes GSTM3, GSTA4, GSTO2 and GSH. GSTs are involved in metabolism and detoxification of electrophilic compounds by conjugation with glutathione. Enrichment in aminoacyl-tRNA biosynthesis, metabolism of xenobiotic and cysteine and methionine metabolism was also observed (Fig. 4E, Supplementary Table 3).

Among the downregulated DEGs, we recognized enrichment in inositol phosphate metabolism (n.Hits=12, adj.p value = 1.35e-09), which is indicative of the regulation of intracellular cation homeostasis, glutathione metabolism and central carbon metabolism (Fig. 4F, Supplementary Table 3).

Finally, we extracted the lists of genes that hit specific pathways potentially involved in metabolic rewiring and distinct mechanisms of cell death. These data further support heterogeneous outcomes across different pathways and not a unique targeting effect (Supplementary Fig. 4).

#### DMF reduces the TLR-induced inflammatory and metabolic activation of CLL cells

**Fig. 5:**
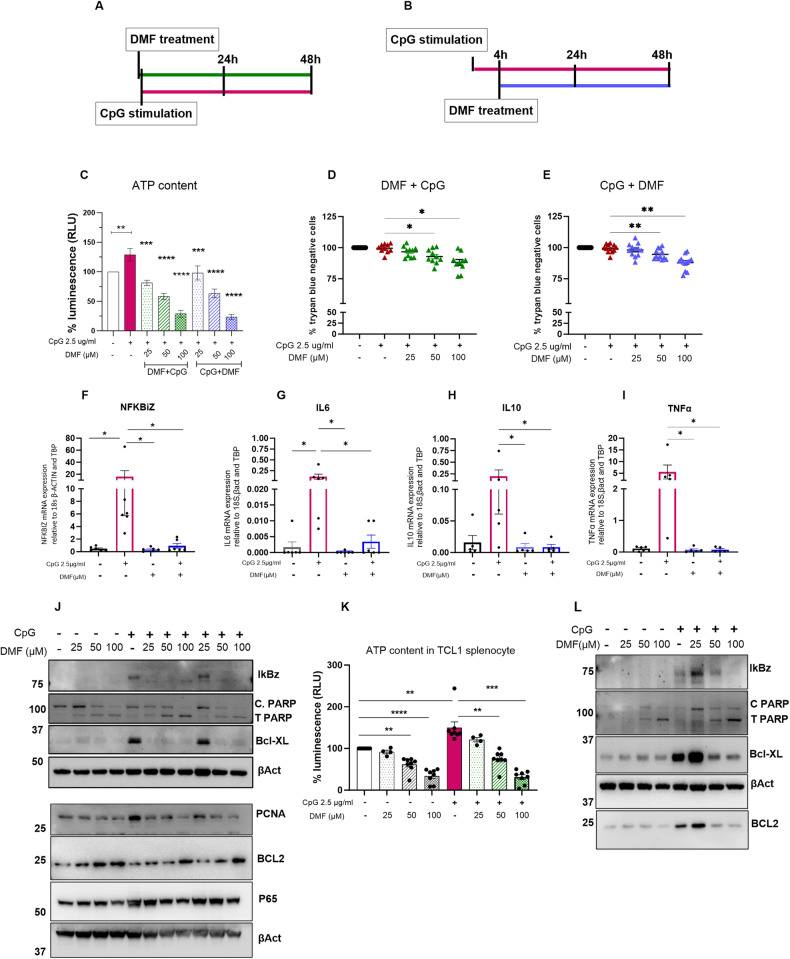
DMF reduces inflammatory stimuli due to TLR9 stimulation with CpG. Time schedule of treatments. Cells were treated with DMF and immediately afterwards with 2.5 μg/ml CpG (**A**) and/or were stimulated for 4 h with CpG, after which increasing concentrations of DMF were applied (**B**). **C** ATP content, a measure of metabolic activation, was addressed by a cell titer assay in CLL cells (*n* = 23) cotreated with DMF and CpG (green bars; 24 h) or pretreated with CpG followed by DMF (blue bars; 24 h). Cell viability was measured in CLL cells cotreated with DMF and CpG (**D** *n* = 10) or pretreated with CpG followed by DMF (**E** *n* = 12). The data are expressed as the mean ± SEM. **p < 0.01, ***p < 0.001. **F**–**I** NFKBIZ (*n* = 6), IL6 (*n* = 6), IL10 and TNFα (*n* = 5) mRNA expression relative to that of 18S, β-actin and TBP was measured by qRT-PCR after 4 h of treatment with 100 µM DMF and/or CpG. The data are expressed as the mean ± SEM. *p < 0.05. **J** PARP cleavage and PCNA protein levels in CLL cell extracts after exposure to DMF and CpG for 24 h were analyzed by western blotting. One representative experiment out of 3. IkBz, BCL-xl, BCL2 and p65 were also analyzed; one representative blot out of 2 is shown. β-Actin was used as a loading control. **K**, **L** ATP quantification and protein expression were evaluated in murine leukemic cells incubated with increasing concentrations of DMF alone or in combination with CpG. **K** Relative ATP content was measured by a cell titer assay in TCL1-transgenic splenocytes (*n* = 8) cotreated with DMF and CpG for 24 h in vitro. **L** IkBz, BCL-xl, BCL2, total and cleaved PARP were analyzed by western blot in leukemic TCL1-transgenic mouse-derived splenocytes cotreated with DMF and CpG for 24 h. One representative experiment out of 2.

To recapitulate in vitro the inflammatory environment mimicking the biological situations occurring in vivo in the lymph nodes and to establish an experimental condition that induces chemoresistance [11], we stimulated primary CLL cells with the TLR9 ligand CpG for 4 h, and subsequently, we added increasing amounts of DMF; in a parallel set of experiments, the same samples were treated with DMF and concomitantly stimulated with CpG (see Fig. 5A, B for a schematic representation). After 24 and 48 h, we measured the capacity of DMF to block CpG-mediated cell activation and survival. We confirmed under both experimental conditions that DMF reduced ATP levels and cell viability in a dose-dependent manner (Fig. 5C–E and Supplementary Figure 5C–E for 24 and 48 h, respectively) and that CpG did not rescue the effect of DMF.

To analyze the molecular pathways involved, we measured the mRNA levels of classic TLR9-induced genes before and after DMF and CpG cotreatment for 4 h. DMF (100 μM) significantly decreased CpG-induced NFKBIZ transcript levels (Fig. 5F; *n* = 6 cases), as did the downstream targets IL6, IL10 and TNFA (Fig. 5G-I). Moreover, DMF blocked CpG-induced IkBz and p65 expression at the protein level and induced PARP cleavage even in the presence of TLR9 agonist. TLR9 stimulation increased PCNA protein expression, confirming the induction of CpG-mediated proliferation, but DMF restored the basal levels of PCNA. BcL-XL was induced by CpG but downregulated by DMF; somewhat surprisingly, BCL2 tended to increase when the cells were incubated with DMF with or without CpG (Fig. 5J and Supplementary Fig. 6).

To further confirm the activity of DMF in an additional preclinical model, murine leukemic splenocytes isolated from TCL1-transgenic leukemic mice were treated in vitro with increasing doses of DMF alone or in combination with CpG. Again, we observed metabolic activation in the presence of TLR9 stimulation that was reduced by DMF co or post treatment (Fig. 5I). Similar to our findings in patient samples, we observed PARP cleavage and BcL-XL decrease after DMF exposure even in the presence of CpG; the IkBz protein was induced by low doses of DMF but reduced at higher doses in 1 out of 2 samples analyzed. In contrast to patient samples, DMF inhibited CpG-mediated BCL2 induction (Fig. 5L).

## Discussion

The aim of this project was to evaluate the potential therapeutic activity of the electrophilic drug dimethyl fumarate in preclinical models of CLL, including cell lines, murine splenocytes and primary leukemic cells isolated from the peripheral blood of patients. We hypothesized that DMF could have a therapeutic effect in vitro based on its well-known immunoregulatory activity and the fact that CLL cells rely on these same regulatory pathways for survival. Moreover, DMF may induce lymphopenia when administered for the treatment of patients with multiple sclerosis [50]. We demonstrated the capacity of this compound to act as an anti-inflammatory, pro-apoptotic drug in primary leukemic cells. At a concentration of 100 μM, DMF reduced cell viability in all the samples analyzed independent of the mutational status of the IGHV genes; however, at lower concentrations, DMF was more active on mutated samples. Similarly, DMF appeared to be less active in the PCL12 cell line, which has unmutated IGHV. Notably, no toxic effect was observed in normal leukocytes isolated from healthy donors, where it is mainly an antioxidant.

Under normal conditions, NRF2 is associated with Keap1, and during oxidative stress, it translocates to the nucleus activating ARE elements. Previous observations showed a controversial role for NRF2 activation in CLL cells. NRF2 activation was shown to exert a protective effect on CLL cells characterized by high ROR1 expression levels and resistance to BCL2 inhibition [51]. In this context, a signaling network through the NF-kB and NRF2 axes may protect leukemic cells from cell death induced by ROS-inducing drugs. On the other hand, previous findings suggest that NRF2 activation causes toxicity in CLL cells [28]. Our present results confirm and extend the latter observations and support the hypothesis that the concomitant inhibition of the NF-kB family and prolonged activation of NRF2 together with its target HO-1 may shift the balance toward CLL cell death. Moreover, RNA-seq analysis further supported the evidence that DMF blocks typical inflammatory pathways and NF-kB-related signatures.

Regarding the molecular mechanisms involved in the observed toxicity, we first analyzed the levels of GSH, a known target of DMF in other cellular contexts [29]. We demonstrated that DMF depletes GSH levels in the MEC1 cell line and primary CLL cells at 4 h, leading to complete loss after 24 h. In contrast, in healthy PBMCs, we observed only a slight decrease, suggesting possible selectivity toward CLL cells where the GSH pool may not be recycled, leading to cell death. In fact, apoptosis was induced, as demonstrated by Annexin V exposure and caspase-3 and PARP cleavage; this was accompanied by a decrease in Bcl-XL but not in BCL2.

These data indicate that the depletion of GSH (which usually induces oxidative stress) is followed by the activation of NRF2 (which is a key antioxidant transcription factor) and eventually results in apoptosis. Surprisingly, no increase in ROS was detected under these experimental conditions, suggesting that prolonged NRF2 activation may dampen ROS production but may not be sufficient to protect from cell death.

DMF strongly reacts with GSH as well as with other proteins containing reactive cysteines, resulting in the succination of several targets. Using NAC (N-acetylcysteine), an antioxidant agent and precursor of cysteines that can increase GSH levels [52], we completely restored the ATP content and dampened DMF-mediated toxicity, further suggesting that GHS depletion may be the main mechanism of action of DMF in CLL cells.

Previous studies reported different forms of cell death due to DMF treatment, including ferroptosis, in lymphoma [46]; in this work, we examined the induction of lipid peroxidation and LPCat3 expression, which are typical readouts of ferroptosis and seem not to be involved in CLL cell death following DMF treatment. However, some patients show lipid peroxidation, and these same cases are sensitive to iron (FeAC).

Monomethyl fumarate (MMF) is considered the active metabolite of DMF in vivo mainly because DMF is undetectable in the serum after oral administration [29–31]; however, DMF-GSH adducts can be found in the urine of patients [53] and circulating in rodents’ plasma, portal vein blood and brain [47, 54]. Moroever, Dimethyl succinated GAPDH can be found in mouse tissues (spleen and brain) [38]. These data support that not all DMF is hydrolyzed to MMF in the intestine and that DMF may enter the circulation to target different cell types. Our data showed that MMF was less active than DMF as a pro-apoptotic agent in vitro. Accordingly, several studies previously reported a less pronounced activity of MMF compared to DMF in vitro [36, 37, 41]. Based on these obervations, future preclinical trials with both DMF and MMF in mouse models of CLL will be instrumental for optimizing treatment options.

Patients with CLL are currently managed with signaling inhibitors such as BTKi and proapoptotic BCL2 inhibitors, and novel combinations are being tested in refractory cases. In this context, we tested DMF in combination with second-generation BTKi acalabrutinib or venetoclax; a mild and heterogeneous synergism emerged with the BTK inhibitor only. Looking at a possible molecular mechanism, we observed that BCL2 was not inhibited by DMF; rather, a trend toward an increase was observed in primary CLL cells, as previously reported in other cellular contexts [55].

These data highlight the importance of identifying novel combination strategies and suggest the potential use of DMF with signaling inhibitors. DMF is a drug currently used for the treatment of psoriasis and multiple sclerosis [30]; an ongoing clinical trial was recently designed to test DMF in cutaneous T-cell lymphoma [41, 56], suggesting its potential use in lymphoid malignancies. Similarly, several preclinical studies proposed the use of DMF in different types of leukemia and solid tumors: DMF was active in AML differentiation therapy [57], inhibited melanoma growth and metastasis [58–60], blocked constitutive NF-kB signaling in breast cancer cells resulting in apoptosis [61, 62], and repressed tumor progression in non-small cell lung cancer models [63]. However, our study is the first to focus on DMF-induced cell death in CLL where it may target a novel vulnerability in the disease towards an actual clinical translation.

## Materials and methods

### Reagents and chemicals

DMF (cat. no. S2586) was purchased from Selleckchem (Houston, TX, USA) and from DBA (HY-17363, Med Chem Express), MMF from DBA (HY-103252S, Med Chem Express), acalabrutinib (cat. HY-17600) from DBA, a BCL-2 inhibitor, venetoclax from BioVision (Waltham, MA, USA), navitoclax (ABT-263, cat. HY-10087, Med Chem Express) from DBA and fludarabine phosphate from Selleckchem. The human TLR9 ligand CpG oligonucleotide (ODN2006) and the murine-specific CpG oligonucleotide (ODN1826) were purchased from InvivoGen (San Diego, California, USA). Ferric ammonium citrate (FeAC, cat 1185-57-5) and N-acetyl-L-cisteina (NAC, cat A8199) from Sigma‒Aldrich (St. Louis, MO, USA). Sterile DMSO was purchased from Sigma‒Aldrich. All drugs were dissolved following the manufacturer’s instructions, and we selected the appropriate vehicle in untreated cells (NT) used as experimental control.

### Patients

Heparinized peripheral blood was obtained from patients after providing informed consent, and the study was conducted as part of a study approved by the institutional ethics committee at IRCCS San Raffaele Hospital (IRB approval code VIVI-CLL). All patients with CLL selected for the study were diagnosed according to the International Workshop on CLL 2018 Guidelines [64] and were off therapy or untreated for more than 6 months before this study (Supplementary Table 1 and all the figures except Fig. 2I), except for 5 patients who were under therapy with the BTK inhibitors ibrutinib or acalabrutinib (Supplementary Table 2 and Fig. 2I). The following parameters were analyzed for each patient: age, sex, disease stage, CD38 expression, IGHV gene mutational status, and response to DMF at 50 and 100 µM after 24 h (Supplementary Table 1).

### Cell isolation, in vitro culture, and treatment

CLL B cells were negatively selected and purified from (*n* = 65) patient total peripheral blood mononuclear cells (PBMCs) and cultured as previously described [11]. PBMCs from healthy donors were isolated by Ficoll (IRB approval code Leu-Buffy_Coat). The MEC1, MEC2, HG3 and PCL12 cell lines were purchased from DSMZ, cell bank (Cell Bank of the Deutsche Sammlung von Mikroorganismen und Zellkulturen GmbH, Braunschweig, Germany) and cultured in RPMI 1640 medium (Euroclone, Pero, Milano) supplemented with 10% heat-inactivated FBS and Pen-Strep (1000 U; Sigma‒Aldrich St Louis, MO, USA) at a concentration of 1 × 10^6^ cells/ml for early treatments and at 8 × 10^5^ for longer treatments. All cell lines were routinely tested for mycoplasma contamination.

CLL cells, leukemia cell lines and PBMCs from healthy buffy coats were treated with increasing concentrations of DMF (from 6.25 to 100 µM) in the presence or absence of CpG (2.5 μg/ml). CpG was added 4 h before DMF or immediately after DMF addition (Supplementary Fig. 3). MEC1 and CLL cells were treated with 3 mM NAC w/o DMF for 4 or 24 h.

### Cell viability and cell cytotoxicity assays

The samples were analyzed for metabolic activation, which reflects cell viability, by measuring the ATP content using the Cell Titer-Glo assay (Promega, Madison, WI, USA) according to the manufacturer’s manual. Twenty-four, 48, and 72 h after treatment, luminescence was measured using Viktor (Perkin Elmer). To measure cell viability and determine the proliferation rate, CLL cells or cell lines were collected at the same time points and counted with the automatic counter TC20 with trypan blue dye (Bio-Rad, Hercules, CA, USA). To assess cytotoxicity, CellTox™ Green Cytotoxicity Assay (Promega) was used according to the manufacturer’s manual. The resulting fluorescence signal produced by the binding interaction with dead cell DNA is proportional to the cytotoxicity.

### Flow cytometry

Apoptotic cell staining was performed with an Annexin V-FITC and propidium iodide kit (e-Bioscence, San Diego, CA) in *n* = 8 primary CLL samples following the manufacturers’ protocol.

Healthy PBMCs were stained with a viability dye (780 Invitrogen, e-Bioscience) and anti-CD5 PC7 and anti-CD19 ECD antibodies (Beckman Coulter, Brea, CA, USA). To detect the presence of lipid oxidation indicating ferroptosis, CLL cells were stained in Hank’s balanced salt solution (HBSS) for 10 min at 37 °C with 5 μM C11-BODIPY 581/591 (#27086, Cayman Chemical, Ann Arbor, MI, USA), and the mean fluorescence intensity was detected by flow cytometry.

To investigate the presence of reactive oxygen species (ROS), CLL cells were stained with viability dye and with the cell-permeant 2′,7′-dichlorodihydrofluorescein diacetate (H2DCFDA) (D399, Invitrogen, Thermo Fisher Scientific, Waltham, MA, USA) by diluting the reagents to a final working concentration of 1 µM in prewarmed PBS and incubating for 15 min at RT.

All experimental data were acquired with BD Navios flow cytometer (BD Pharmingen), and the results were analyzed using FCS Express 7 software (De Novo software).

### IC50 calculation and combination studies

Cell viability data obtained from the CellTiter-Glo assay, expressed as the percentage of treated versus untreated cells, were used to calculate the IC50 with GraphPad Prism. We calculated the average metabolic cell activation at different dosage combinations with respect to untreated sample concentrations. DMF was combined with acalabrutinib (*n* = 8), venetoclax (*n* = 6) or navitoclax (*n* = 5), and the data were analyzed by the Synergy Finder web application for interactive analysis and visualization of drug combination screening [43]. The software is available at the following website: https://synergyfinder.fimm.fi. The following parameters were obtained: IC50 (concentration that causes 50% inhibition), combination index (CI) and synergy score. A synergy score <−10 indicates an antagonistic effect, while a synergy score >10 indicates synergism; values between −10 and 10 indicate negative or positive additivity.

### RNA sequencing

RNA sequencing of CLL patient cells treated with 100 µM DMF and untreated of 5 different experimental pairs was performed at the Genomics Laboratory of CORS (Center for Omics Science), Ospedale San Raffaele. The samples used are marked with * in Supplementary Table 1. Gene expression analysis was performed to compare treated samples to controls. Differential gene expression analysis was performed using limma [65]. Genes were considered “differentially expressed” if they satisfied both the following conditions: nominal p value < 0.01 and absolute value of log_2_ FC ≥1. The raw data were deposited in the Gene Expression Omnibus (GEO) at GSE245432. Volcano plots were generated with the R tool using log_2_FC and P values of DMF-treated samples versus untreated samples. Heatmaps were created with Heatmapper [66] using Log2RPKM values. Further enrichment analyses were performed on the lists of DEGs with the Metascape online tool (https://metascape.org), and tabular data were sorted for combination score and p value > 0.005 [49]. The custom analysis allowed us to explore the most differentially expressed TFs involved in DMF activity. Integrative analysis of genes and related metabolite molecular interactions was performed with the OmicsNET 2.0 tool (http://omicset.ca) using a Kyoto Encyclopedia of Genes and Genomes (KEGG)-based metabolic network.

### RNA extraction, cDNA synthesis and real-time PCR analysis

Total RNA was extracted from CLL cells and from splenocytes using a Mini kit following the manufacturer’s instructions (Qiagen, Hilden, Germany). cDNA was synthesized from 0.5 μg of total RNA with an Iscrip kit (Bio-Rad). To detect mRNA levels of target genes, quantitative RT‒PCR was performed using SsoAdvanced™ Universal Probes Supermix and the following commercial probes labeled with FAM or HeX dye (cat. 12001950, Bio-Rad): NFKBIZ (qHsaCEP0052487), NQO1 (qHsaCEP0039593), β-Actin (qHsaCEP0036280), MRPS18A (qHsaCEP0049956), TBP (qHsaCIP0036255), and Applied Biosystems assays-on demand IL-6 (Hs00985639), IL10 (Hs00961622), TNFα (Hs00174128), (Thermo Fisher Scientific, Waltham, MA, USA) or SsoAdvanced™ Universal SYBR Green Supermix (Bio-Rad) and the primers listed below: GPX4 (Fw -5′-TGG GAA ATG CCA TCA AGT-3′, Rev 5′-GGG GCA GGT CCT TCT CTA TC-3′), LPCAT3 (Fw 5′-GGT AGA AAA GGC CCA GAC TCA-3′, Rev 5-ACC AGG AAA GAT ACC AAA CAG C-3), NRF2 (5′-CAGCGACGGAAAGAGTATGA-3′ 5′-TGGGCAACCTGGGAGTAG-3′) and B-actin qHsaCED0036269. Real-time PCR was performed using CFX Connect Real-Time PCR Detection System with Probe protocol. Relative mRNA expression was calculated as 2^-Dct “gene of interest”/beta-actin, 18S, TBP, as indicated in the figure legends.

### Western blot analysis

Lysates (RIPA buffer) were diluted in NuPAGE LSD Sample buffer and sample reducing agent (Invitrogen) and run on reducing SDS‒PAGE mini-gels (Invitrogen).

Proteins were electrotransferred onto nitrocellulose membranes with a turboblot (30 V, 7 min) and then incubated overnight at 4 °C with the appropriate antibody diluted at the optimal concentration in 5% (w/v) BSA or skim milk. The following antibodies were used for Western blot analysis: BCL2 (Millipore cat. 05-826, Merck), BCL-XL (Cell Signaling Technology, Danvers, MA, USA, 54H6 #2764 1:1000), PARP (CST, cat. 9542, 1:1000), PCNA (CST, cat. 13110, 1:1000), NFKBIZ (CST, cat. 9244 1:1000), NF-κB p65 (CST, cat. 8242 1:1000), Survivin (CST, cat. 2808 1:1000), NRF2 (cat. ARG40635 Arigo, Hsinchu City, Taiwan 1:1000), NQO1 (cat. ARG43340,Arigo 1:1000), HO-1 (cat. ARG43341, Arigo 1:1000), GPX4 (ARG41400, Arigo 1:1000), Laminin B1-HRP conjugate (CST, cat. 15068 1:1000) and anti-β-Actin HRP-conjugated (Sigma 1:20000). The blots were then washed in PBS with 0.2% Tween (PBST) and incubated with the appropriate HRP-conjugated secondary antibody for 1 h at RT. The proteins were visualized using the enhanced chemiluminescence reagent ECL Clarity (Bio-Rad) and detected with a ChemiDoc imaging system (Bio-Rad).

### GSH assay and TransAM NRF2

The levels of reduced glutathione (GSH) were detected with a GSH/GSSG-Glo assay (V6611, Promega) in primary CLL cells and MEC1 cells (4 and 24 h) and in healthy PBMCs (24 h) according to the manufacturer’s instructions. NRF2 activation was monitored in nuclear extracts (10 µg) obtained with a nuclear extract kit (cod. 40010 Active Motif, Waterloo, Belgium) from DMF-treated (50 and 100 µM for 4 h) and untreated MEC1 cell lines with the TransAM NRF2 kit (cod.502960 Active Motif) following the manufacturers’ instructions. The data are the results from one experiment assayed in technical duplicate.

### Ex vivo experiments with TCL1-transgenic mouse splenocytes

The percentage of leukemic cells was monitored in the peripheral blood of TCL1-mice (*Mus musculus*, C57BL/6 Tcl-1 transgenic; 8 adult mice, 3 females and 5 males) by flow cytometry of circulating cells stained with anti-CD45 APC, anti-CD19 PE-Cy7 and anti-CD5 PE (BioLegend Inc., San Diego, CA). Mice were sacrificed when the percentage of leukemic cells circulating in the peripheral blood exceeded 70% (IACUC number 716). The spleens were collected, and spleen-derived splenocytes were isolated and cultured in RPMI 1640 medium supplemented with 10% heat-inactivated fetal bovine serum (FBS), 1% sodium pyruvate, 1% HEPES, 50 μM β-mercaptoethanol and 1% penicillin‒streptomycin at a density of 3 × 10^6^ cells/ml in a humidified atmosphere at 37 °C containing 5% carbon dioxide. Increasing concentrations of DMF alone or with CpG were applied to splenocytes.

### Statistical analysis

Statistical analyses were performed with GraphPad Prism software Version 10.02 (La Jolla, CA, USA) as indicated in each figure legend. Probability values < 0.05 were considered statistically significant. To compare two groups of samples, a two-tailed t test was performed considering a 95% confidence interval (CI) with an alpha error of 0.05. For more than two groups, one-way or two-way ANOVA followed by Dunnett’s multiple comparison post hoc test was used for normally distributed populations to compare each of the treatment groups. The data are presented as the mean ± SD or mean ± SEM, as indicated in each figure legend.

The sample size of 46 patients was calculated using G*power software (Universität Düsseldorf, Germany) considering a specific power of 0.8 with an alpha error of 0.05 and to observe differences with an effect size of 0.8 [67, 68].

### Reporting summary

Further information on research design is available in the Nature Research Reporting Summary linked to this article.

### Supplementary information

Supplementary information

Original data file

Reporting Summary

## Data Availability

The RNAseq data that support the findings of this study are openly available in Gene Expression Omnibus at [https://www.ncbi.nlm.nih.gov/geo/], reference number GSE245432. The other data that support the findings of this study are available from the corresponding author upon reasonable request.
