# Supplementary material for: Disrupting pro-survival and inflammatory pathways with dimethyl fumarate sensitizes chronic lymphocytic leukemia to cell death
Source: Cell Death Dis. 2024 Mar 18;15(3):224. doi: 10.1038/s41419-024-06602-z (PMC10944843; doi:10.1038/s41419-024-06602-z)

Mantione et al Supplementary information

Supplementary Table 1. Clinical-biological characteristics of the patients analyzed (off therapy) and percentage of cell viability detected with CTG assay (% ATP content) after 24 hours of 50µM and 100 µM DMF treatment in vitro.

Supplementary Figure 1. Densitometric analysis of western blot results presented in Fig. 1. Signal intensity was normalized to the loading control βactin. Data are shown as mean ± SEM of at least two independent experiments; * indicates p<0.05. Quantification of total PARP (n=3, panel **A**) and cleaved PARP (n=3, panel **B**) and cleaved PARP/total PARP ratio (n=3, panel **C**), cleaved Caspase 3 (n=2, panel **D**), survivin (n=3, panel **E**), PCNA (n=2, panel **F**), P65 (n=4, panel **G**), BCL-2 (n=3, panel **H**) and BCL-XL (n=3, panel **I**) protein expression in MEC1 and MEC2 cell lines treated for 24h with increasing doses of DMF.

Supplementary Figure 2. **(A)** Violin plot showing the distribution of the ATP content in Mutated vs Unmutated IGHV samples, 24h after treatment with DMF 100 µM. **(B)** ATP content in CLL samples (n=4) treated with DMF with or without HS5 stromal cells (feeder layer). **(C)** Cell viability of HS5 stromal cells alone, with or without DMF was monitored as control. Data are expressed as mean ± SD; T-test analysis was conducted. **(D-E)** Comparison between DMF (2 different batches were tested) and monomethyl fumarate (MMF) impact on cell ATP content at 24h (**D**, n=5) and 48h (**E**, n=4) in primary CLL samples. Two-way Anova analysis with Dunnett's post-test was performed. Data are expressed as mean ± SEM and mixed-effect analysis with Dunnett's post-test was performed for multiple comparisons. *p<0.05, **p<0.01; ***p<0.001; ****p<0.0001. **(F-G)** CLL cells were treated *in vitro* with increasing concentrations of DMF plus navitoclax (n=5, panel **F**) or venetoclax (n=6, panel **G**). Metabolic activation was measured by cell titer and relative ATP values used to calculate the combination index; the graphs show no synergism. **(H-K)** Densitometric analysis of western blot results presented in Figure 5 (first 4 lanes). Signal intensity was normalized to the loading control βactin. Data are shown as mean ± SEM of at least 6 independent experiments; * indicates p<0.05. Quantification of BCL-XL (n=6, panel **H**), BCL2 (n=6, panel **I**), cleaved PARP (n=7, panel **J**) and total PARP (n=7, panel **K**).

Supplementary Table 2. Clinical-biological characteristics of the patients analyzed (under therapy with BTK inhibitors).

Supplementary Figure 3. **A)** NRF2 activation was measured in MEC1 cells after DMF treatment with an ELISA based assay in technical duplicate; data are reported as relative optical density (OD). **B)** Western bot of NRF2 and its targets HO-1 and NQO1, and loading controls Lamin B1 and Bactin; CLL cells were treated with DMF and nuclear or cytoplasmic fractions were analyzed as indicated. **C)** Western bot of NRF2 and its target HO-1 in nuclear or cytoplasmic fractions of MEC1 treated with DMF in the presence or absence of NAC (3mM) as indicated. **D-F)** Densitometric analysis of western blot results presented in Figure 3H. Signal intensity was normalized to the loading control βactin. Data are shown as mean ± SEM of 5 independent experiments.

Supplementary Figure 4. Starting from the RNA-sequencing data shown in Figure 5, we created distinct heatmaps for distinct pathways with Heatmapper. Visible clusters are separated with blue lines; the significant differentially expressed genes ($|Log_2FC|\geq1$ and pValue<0.001) are marked in red. We extracted the list of genes that hit each specific pathway from GSEA database: **A)** GOBP_GLYCOLYTIC_PROCESS, Systematic name: M46722. **B**) REACTOME_KEAP1_NFE2L2_PATHWAY Systematic name: M45021. **C**) GOBP_FERROPTOSIS, Systematic name: M46861 and WP_FERROPTOSIS, Systematic name: M39768". **D)** WP_Autophagy, Systematic name: M39904. E)

WP_WARBURG_EFFECT_MODULATED_BY_DEUBIQUITINATING_ENZYMES_AND_THEIR_SUBSTRATES, Systematic name: M48053".

Supplementary Figure 5. Time schedule of treatments. Cells were treated with DMF and immediately afterwards with CpG 2.5 µg/ml (**A**) and/or were stimulated were for 4h with CpG and then increasing concentration of DMF were applied (**B**). ATP content was addressed by in CLL cells (n=18) co-treated with DMF and CpG (green bars) or pre-treated with CpG and following with DMF (blue bars) for 48h (**C**). Cell viability was measured in CLL cells co-treated with DMF and CpG (**D**, n=10) or pre-treated with CpG and following with DMF (**E**, n=12). One-way Anova analysis with Dunnett's post-test was performed. Data are expressed as mean ± SEM. *p<0.05, **p<0.01, ***p<0.001 ****p<0.0001. Measure of cell viability TCL1-transgenic splenocytes (n=8) at 24h (**F**). Relative ATP content measured by cell titer assay in TCL1-transgenic splenocytes (n=8) co-treated with DMF and CpG for 48h in vitro (**G**) and measure of cell viability at 48h (n=8). Data are expressed as mean ± SEM and mixed-effect analysis with Dunnett's post-test was performed for multiple comparisons. **p<0.01; ***p<0.001; ****p<0.0001.

Supplementary Table 3. Integrative analysis of protein-metabolism interaction performed with OmicsNET tool. Top10 enriched pathways emerged from up and downregulated DEGs are listed with total Hits, adjust P value and identifier code for each compound.

Supplementary Figure 6. Densitometric analysis of western blot results presented in Figure 5. Signal intensity was normalized to the loading control βactin. Data are shown as mean ± SEM of 3 or 2 independent experiments (panels A-C and panels D-L respectively).

Supplementary Table1

Sample code	Sex	Surface CD38 result (%)	IGHV Identity	Light chain	Disease status	%ATP content 24h (DMF 50μM vs NT)	%ATP content 24h (DMF 100μM vs NT)
1	M	2.8	93.15	kappa	stable	20.73	14.34
2	F	na	91.67	lambda	stable	63.79	2.23
3	M	0.1	88.42	lambda	stable	19.93	12.83
4	M	0.1	92.28	lambda	stable	na	na
5	M	0.3	100	kappa	stable	88.46	47.44
6	M	0.3	95.79	kappa	stable	58.81	30.46
7	F	18.7	99.66	kappa	stable	75.38	33.07
8	M	0.6	85.61	kappa	stable	na	na
9	M	0.1	98.28	lambda	stable	39.70	13.88
10	M	NA	94	kappa	stable	72.64	37.75
11	M	0.5	92.71	kappa	stable	16.46	13.52
12	M	0	94.39	kappa	stable	na	na
13	M	3.3	100	lambda	progressive	na	na
14	F	0.1	99.65	lambda	stable	104.33	10.58
15	F	0	na	kappa	stable	na	37.99
16	F	na	100	lambda	na	na	2.61
17	F	0.1	100	kappa	stable	16.94	7.45
18	M	5	100	kappa	stable	46.89	12.91
19	M	na	95.09	kappa	na	57.68	21.2
20	M	0.5	95.79	lambda	stable	40.69	24.45
21	F	na	na	na	na	na	na
22	F	na	na	na	stable	41,94	3.37
23 *	M	na	93.06	kappa	stable	na	5.91
24	F	na	90.46	na	stable	51.21	30.1
25	M	0.1	100	lambda	stable	92.09	62.77
26*	M	1.3	100	kappa	progressive	70.28	56.16
27	M	0	91.72	lambda	progressive	20.61	6.98
28	F	na	na	na	stable	84.44	59.36
29*	F	na	90.97	na	stable	62.62	22.07
30	F	na	97.62	lambda	progressive	49.99	1.65
31	M	0.1	100	lambda	stable	74.32	4.99
32	M	0.4	100	lambda	stable	na	8.16
33	F	na	92.63	na	stable	61.67	11.56
34	F	0	95.09	kappa	stable	na	na
35	M	0	na	kappa	stable	na	3.83
36	M	na	96,88	lambda	stable	5.83	1.18
37*	F	na	na	na	na	70.8	37.99
38	F	na	92.6	kappa	stable	na	na
39	M	na	100	kappa	stable	na	na
40*	M	na	95.79	kappa	stable	27.16	15.32
41	M	na	na	na	na	7.19	50.23
42	F	na	na	na	na	34.67	21.69
43	M	na	98.61	lambda	na	na	na
44	F	na	91.93	lambda	na	79.04	39.47
45	F	na	100	kappa	stable	61.66	17.14
46	F	0	na	lambda	stable	18.09	4.68
47	F	0	97.57	lambda	stable	38.45	20.44
48	M	0	96.14	lambda	stable	69.5	8.62
49	M	0.2	94.33	kappa	stable	15.48	5.5
50	M	na	na	lambda	stable	65.02	33.41
51	M	na	99.66	kappa	na	77.56	60.28
52	M	na	96.26	kappa	stable	72.78	8.22
53	M	na	na	na	stable	71.75	21.56
54	F	na	na	na	stable	59.34	19.96
55	F	0	100	kappa	progressive	73.24	35.95
56	F	na	na	lambda	stable	44.65	7.6
57	M	30	na	kappa	stabile	43.54	20.47
58	M	0	98.61	kappa	stable	na	13.54
59	F	0,2	96.84	kappa	stable	64.05	20.06
60	F	0,1	89.47	kappa	stable	39.3	9.48
61	F	na	na	na	na	45.44	11.00
62	na	na	99.65	lambda	progressive	63.49	21.14
63	F	0.57	92.36	lambda	stable	na	na
65	F	44.9	na	kappa	stable	39.8	14.2

Supplementary Figure 1

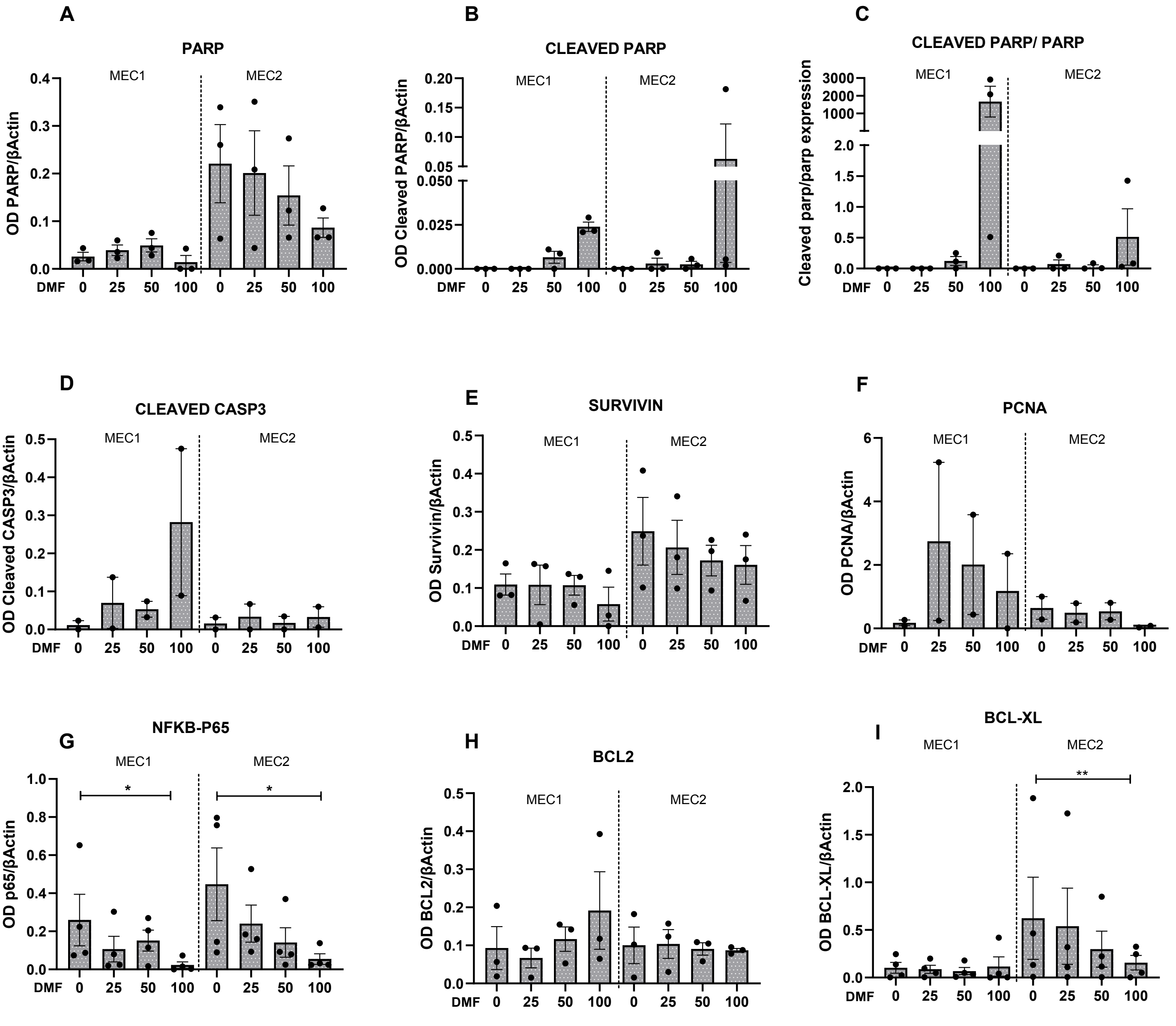

Supplementary Figure 2

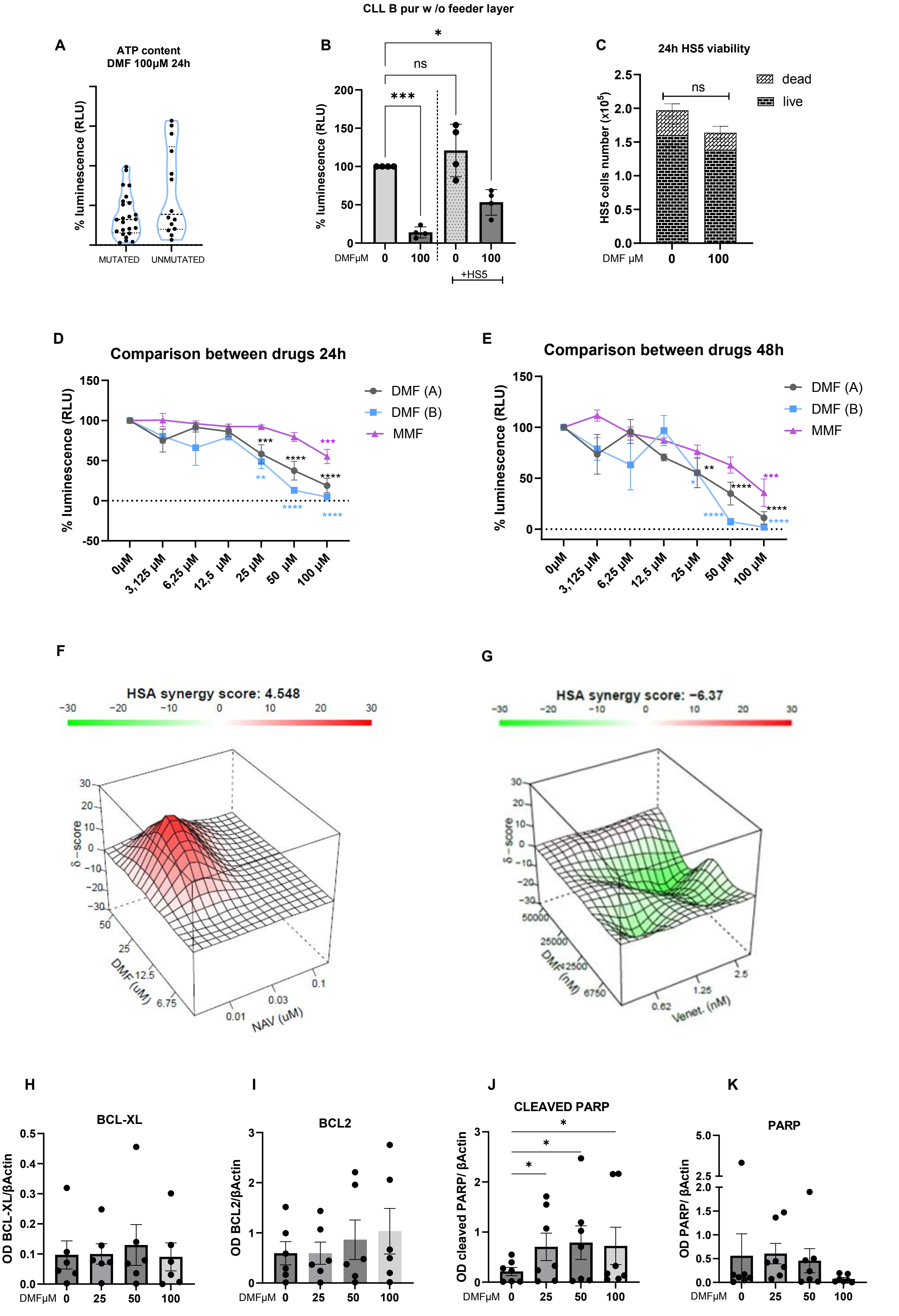

Supplementary Table2

Sample Code	Sex	Surface CD38 result (%)	IGHV Identity	Light chain	Ongoing treatment	% ATP content 24h (DMF 50µM vs NT)	% ATP content 24h (DMF 100µM vs NT)
1	F	5,3	100	lambda	IBRUTINIB	61.51	21.55
2	F	2	90.97	kappa	IBRUTINIB	59.95	46.76
3	M	3,4	98.63	lambda	ACALABRUTINIB	26.06	5.13
4	NA	0,7	100	kappa	IBRUTINIB	32.60	23.83
5	F	2,4	na	lambda	IBRUTINIB	40.78	6.92

Supplementary Figure 3

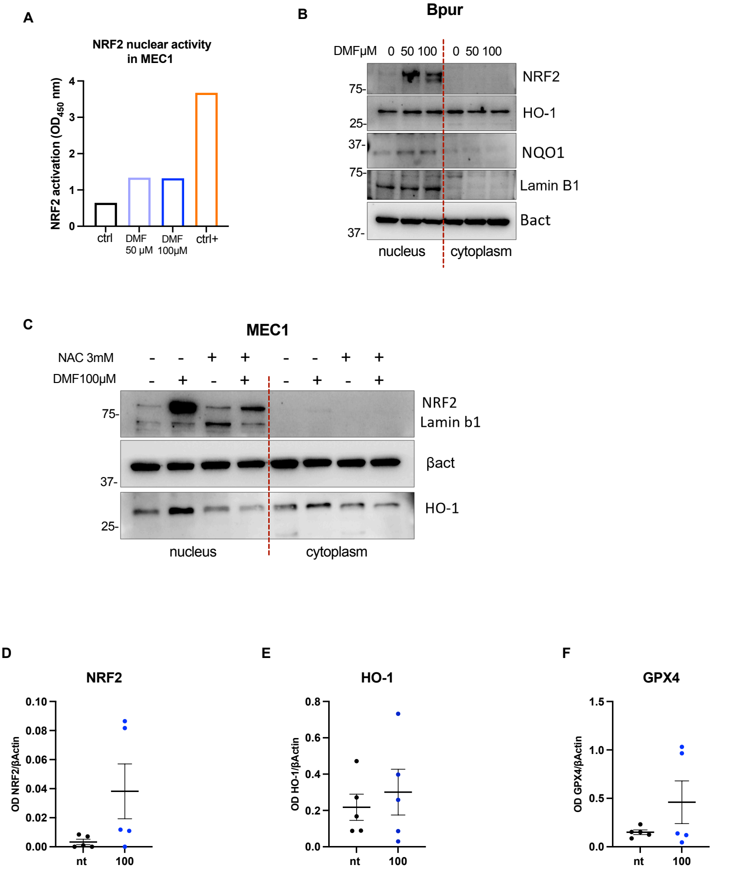

Supplementary Figure 4

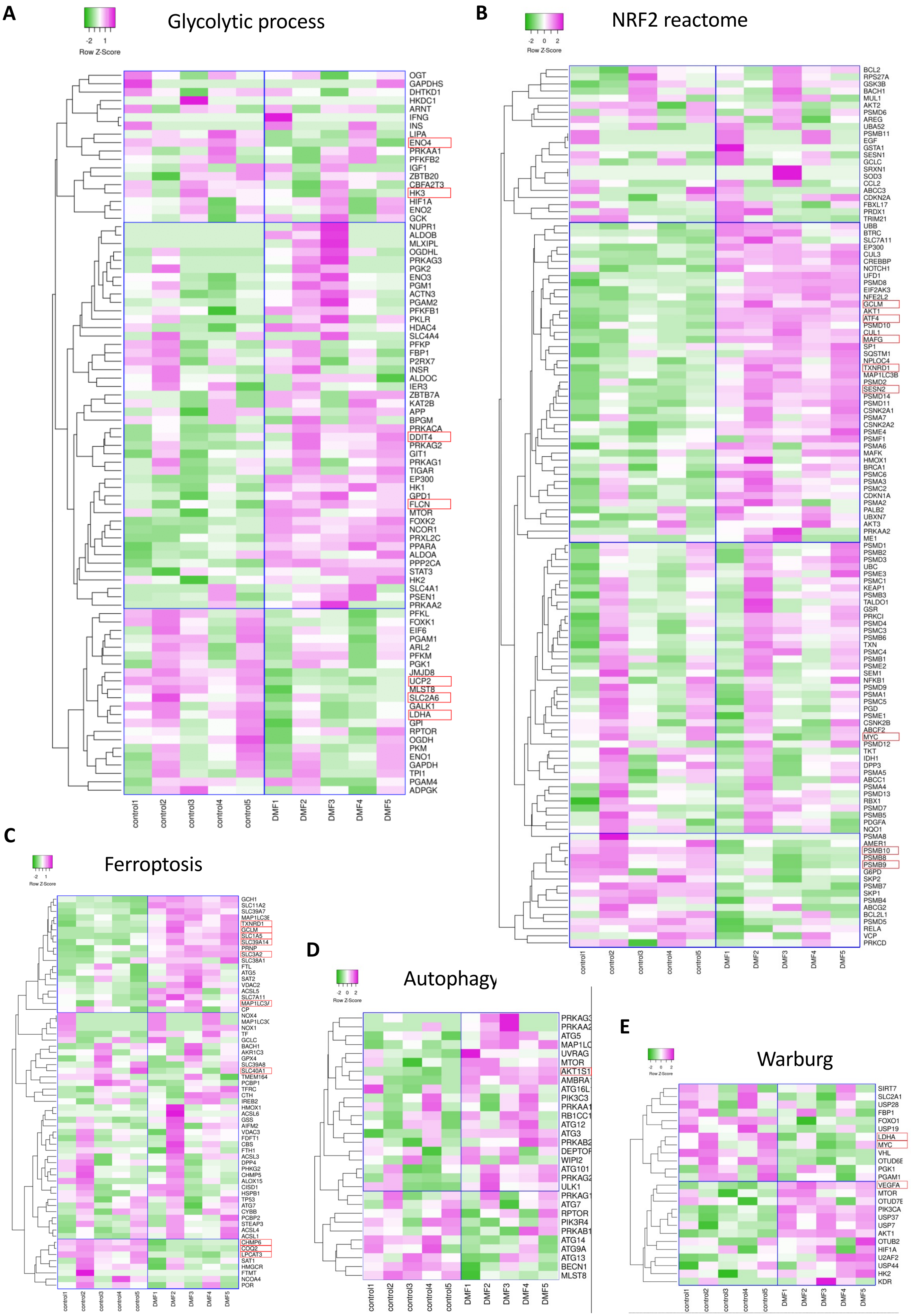

Supplementary Table3

Top 10 Pathway from upregulated Degs	Total hits	integ Pvalue	Features
Metabolism of xenobiotics by cytochrome P450	37	4.79e-30	C14859 C14864 C11088 C14804 C14863 C14793 C14802 C11278 C14800 C14858 C14803 C14856 C14861 C14840 C06790 C14848 C14791 C14792 C14805 C14855 C14839 C14852 C14874 C14847 C14870 C19586 C14865 C14806 C14871 C14857 C14851 C14786 C14868 C14787 2947 2941 119391
Aminoacyl-tRNA biosynthesis	19	4.03e-16	C00148 C01005 C00025 C00065 C02839 C00082 C03125 C00097 C03402 C00152 C00064 C00049 C02987 C02702 C00079 8565 833 4677 2058
Central carbon metabolism in cancer	12	8.52e-11	C00148 C00026 C00025 C00065 C00036 C00074 C00082 C00097 C00152 C00064 C00049 C00079
Cysteine and methionine metabolism	14	2.07e-09	C00051 C01005 C03232 C00065 C00097 C00049 C00606 C00957 C05527 C05528 C00506 C05526 29968 2805
Glutathione metabolism	11	8.24e-08	C00051 C02320 C01322 C00025 C00097 C01419 C01879 2947 2941 119391 79094
Alanine, aspartate and glutamate metabolism	9	1.72e-07	C03912 C00026 C00025 C00036 C00152 C00064 C00049 440 2805
Protein digestion and absorption	10	3.13e-07	C00148 C01327 C00025 C00065 C00082 C00097 C00152 C00064 C00049 C00079
Proximal tubule bicarbonate reclamation	5	6.53e-05	C00026 C00025 C00036 C00074 C00064
Glycine, serine and threonine metabolism	8	0.000118	C01005 C03232 C00740 C00065 C00097 C00049 29968 5723
ABC transporters	13	0.000185	C01157 C00148 C00051 C00025 C00065 C00064 C00049 C00079 5831 29968 5723 440 2805

Top 10 Pathway from downregulates Degs	Total hits	integ Pvalue	Features
Inositol phosphate metabolism	12	1.35e-09	C01204 C01272 C01245 C04637 C00641 C01194 C01220 C01277 C05981 9562 5333 5294
Phosphatidylinositol signaling system	9	3.51e-09	C01204 C01272 C01245 C04637 C00641 C01194 C01220 C01277 C05981
Lysine degradation	12	3.95e-09	C00026 C01149 C01181 C00037 C00047 C00042 C00450 C00956 C04076 224 219 501
Arginine and proline metabolism	13	1.61e-08	C00555 C02946 C00433 C05936 C00334 C00062 C00077 C00581 C01035 224 219 2628 501
Glycolysis / Gluconeogenesis	10	1.59e-07	C00033 C00084 C00074 C00631 C01159 224 219 387712 9562 501
Glycine serine and threonine metabolism	10	2.25e-07	C00258 C00581 C00037 C00631 C00097 C00719 C00576 2628 132158 501
beta-Alanine metabolism	8	1.8e-06	C00555 C00804 C00099 C00334 C05665 224 219 501
Glycerolipid metabolism	9	2.35e-06	C00577 C00162 C00258 C00631 C00641 224 219 132158 501
Glutathione metabolism	8	3.58e-06	C00077 C00037 C05726 C01419 C00097 C05729 C05727 290
Central carbon metabolism in cancer	7	8.63e-06	C00026 C00062 C00037 C00074 C00631 C00042 C00097

Supplementary Figure 5.

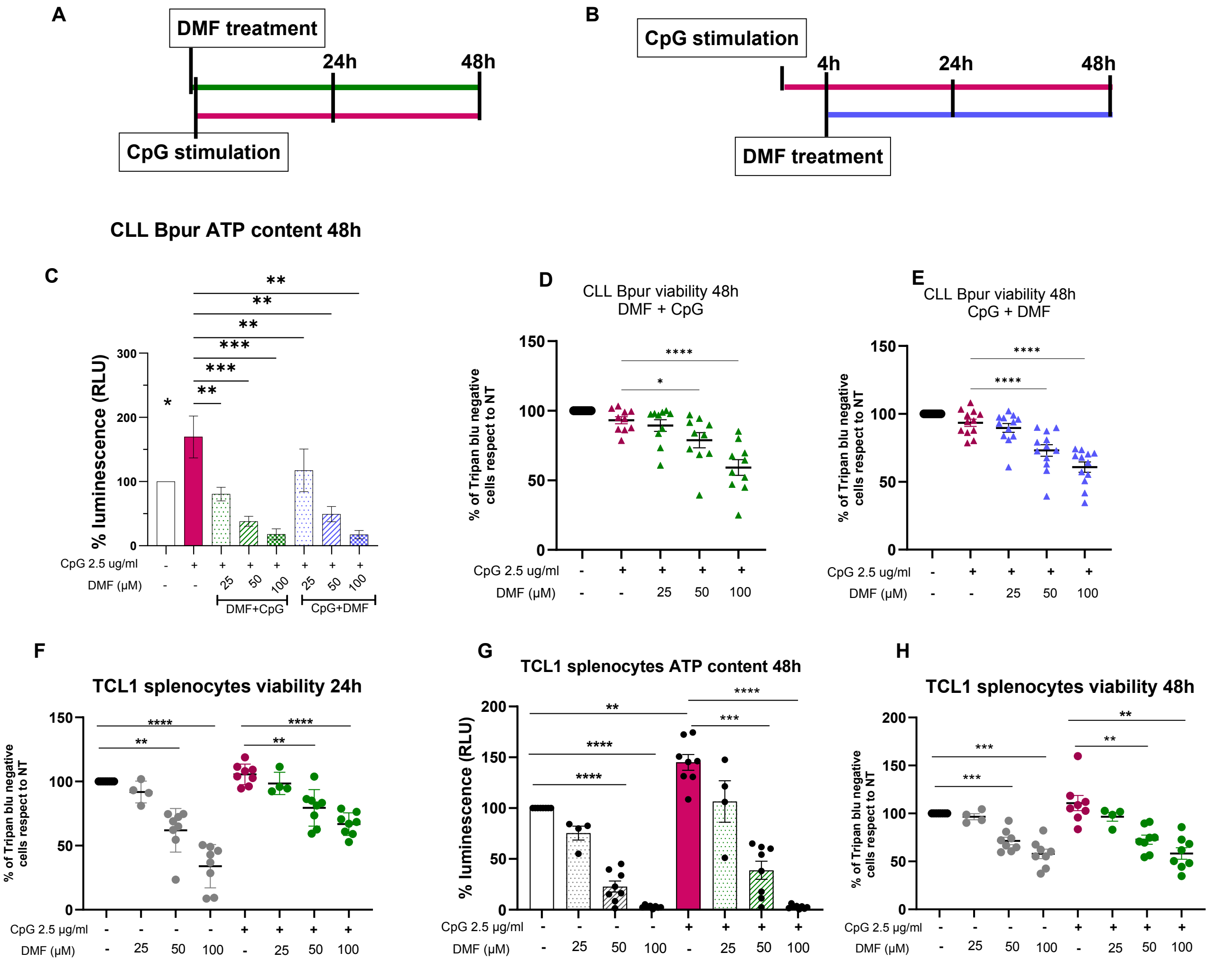

Supplementary Figure 6

Densitometry Bpur: DMF+CpG, CpG+DMF 24h

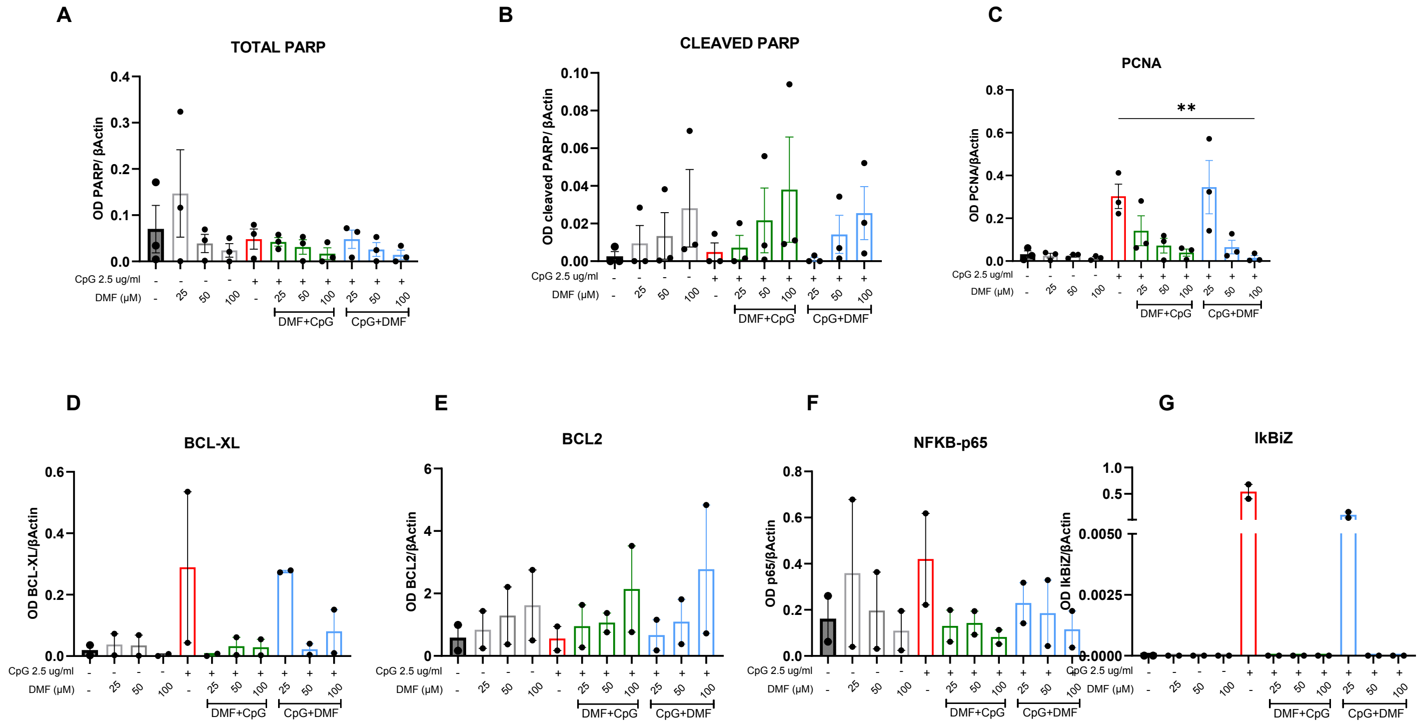

Densitometry splenocytes: DMF+CpG 24h

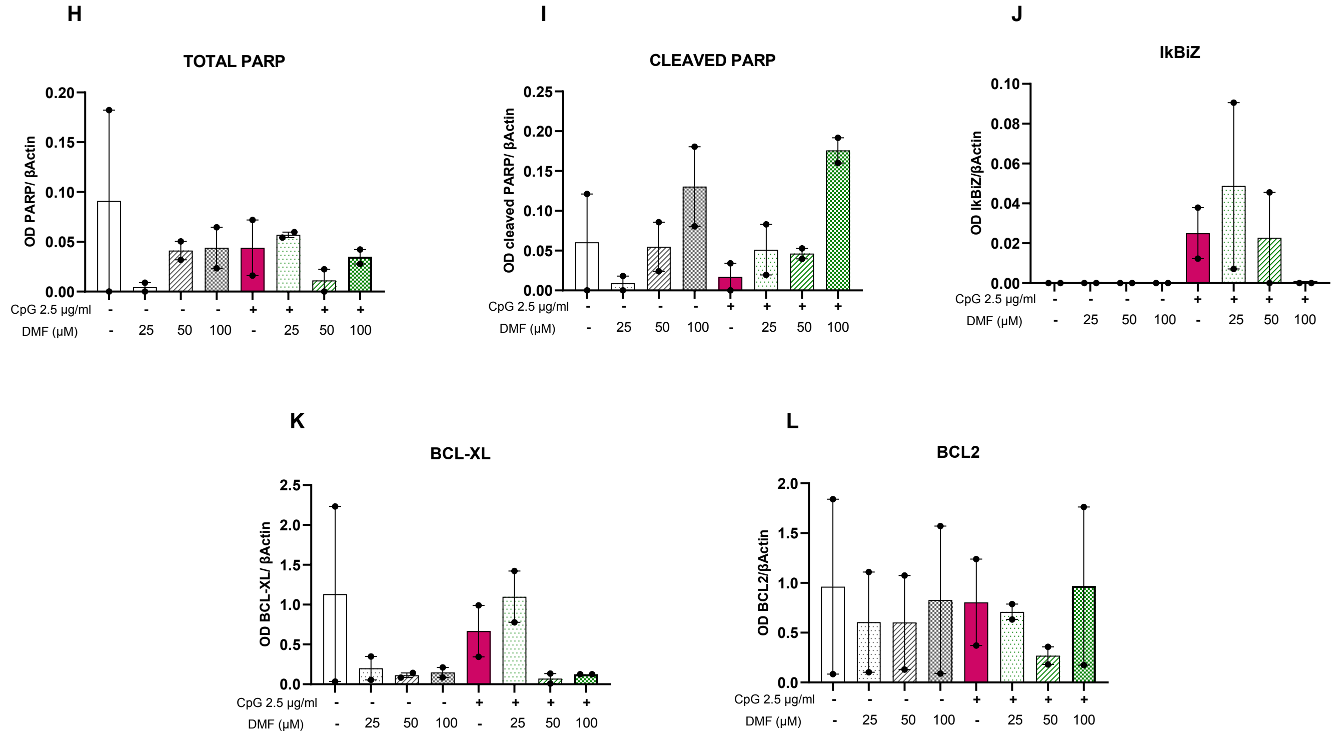

Supplement: Supplementary file 1 — Supplementary information [file 41419_2024_6602_MOESM1_ESM.pdf]
