# Supplementary material for: Disrupting pro-survival and inflammatory pathways with dimethyl fumarate sensitizes chronic lymphocytic leukemia to cell death
Source: Cell Death Dis. 2024 Mar 18;15(3):224. doi: 10.1038/s41419-024-06602-z (PMC10944843; doi:10.1038/s41419-024-06602-z)

Figure. 1-I

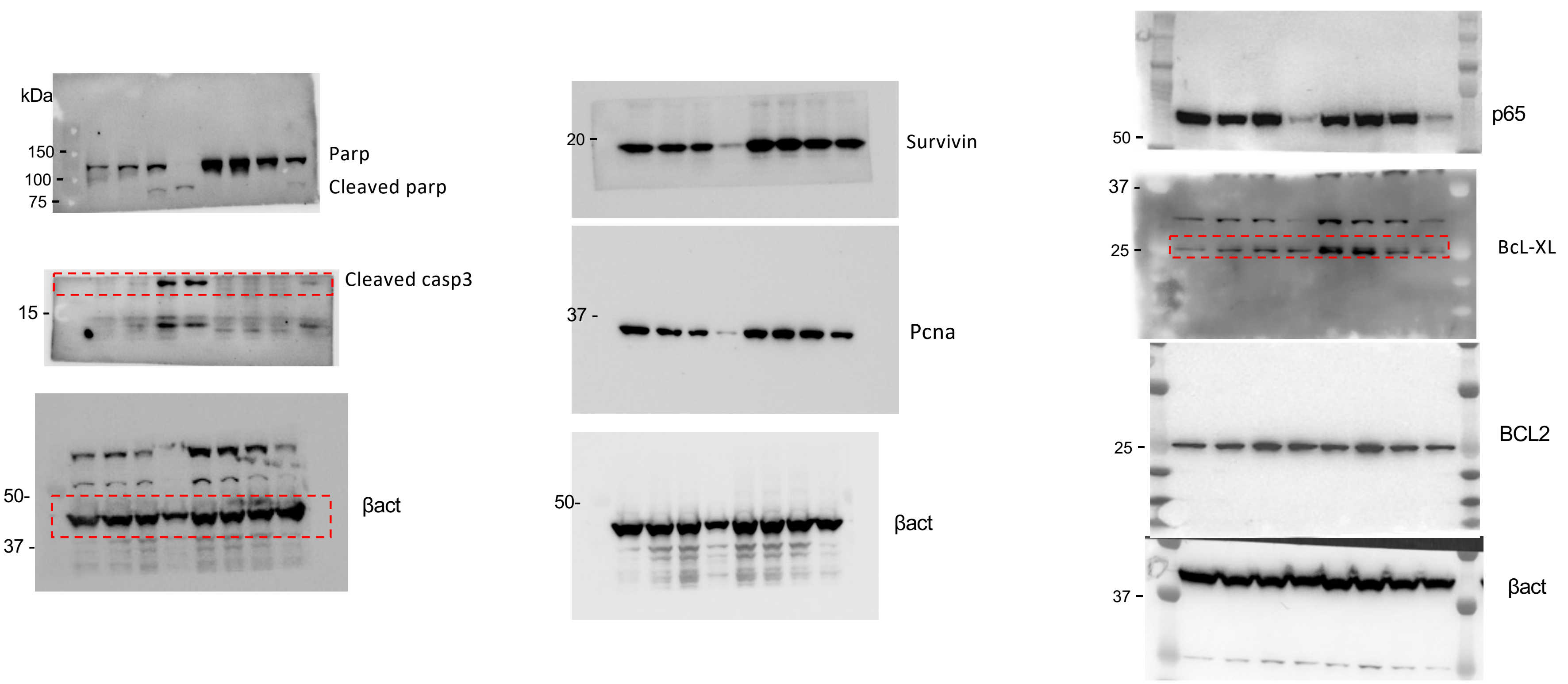
kDa
150
100
75
Parp
Cleaved parp
Cleaved casp3
15
50-
37
βact
20
Survivin
37
Pcna
50-
βact
50
p65
37
25
BcL-XL
25
BCL2
37
βact

Figure 3-H

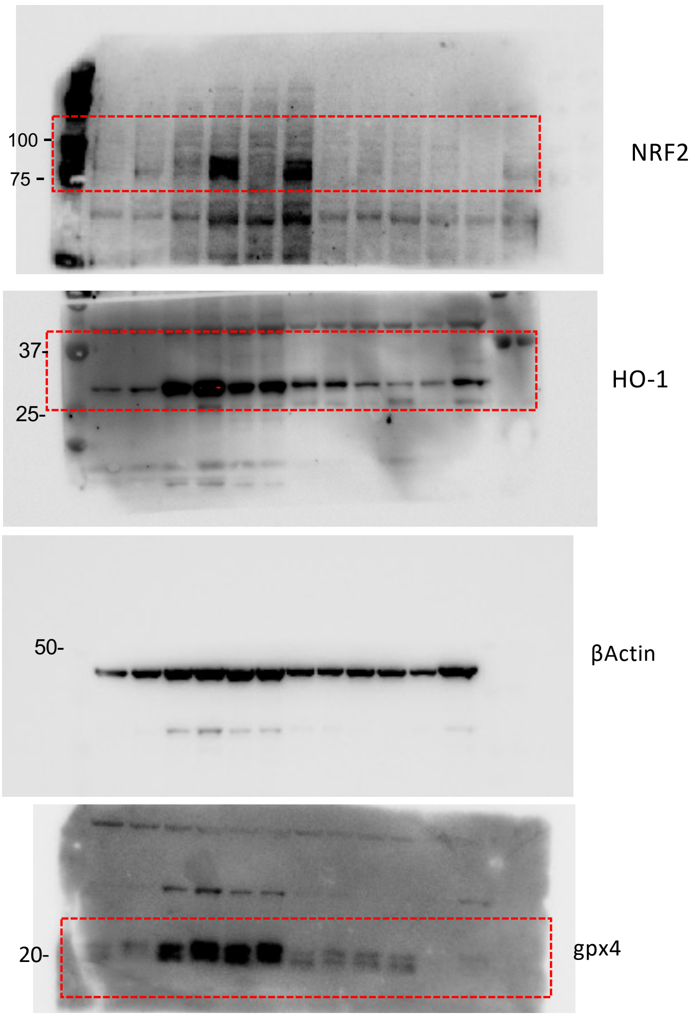

Figure 3-I

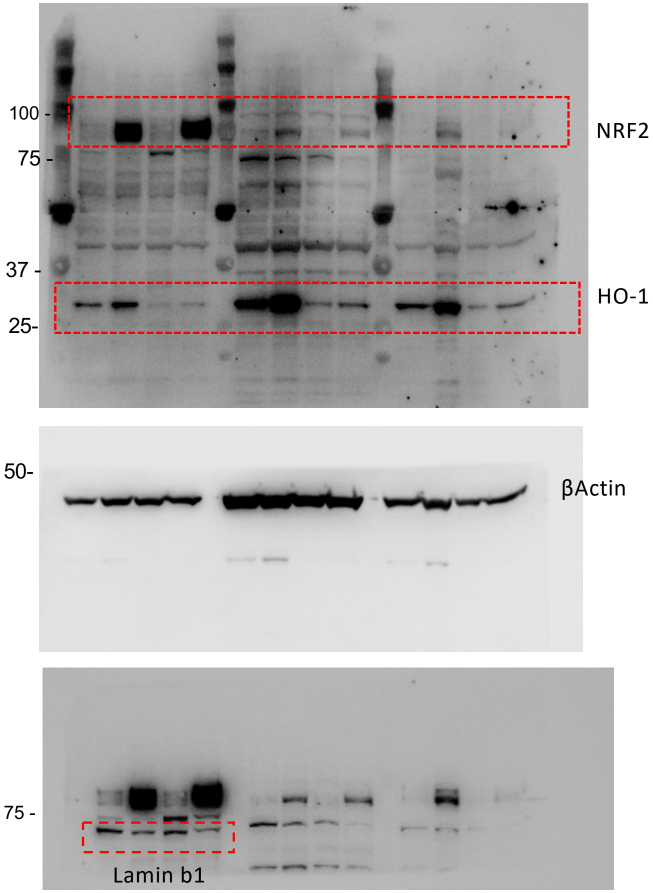

Figure 5J

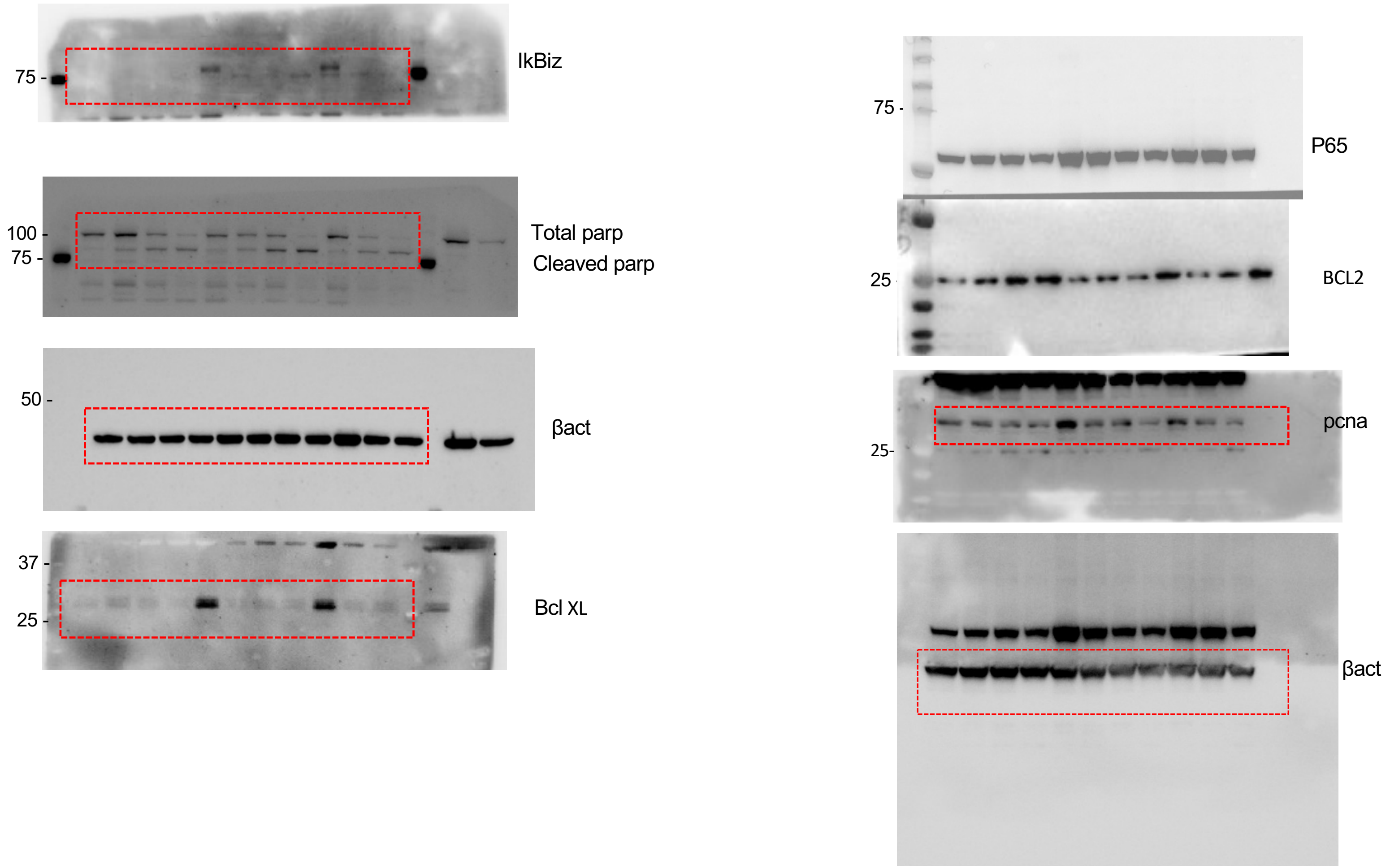
75 -
IkBiz
100 -
75 -
Total parp
Cleaved parp
50 -
βact
37 -
25 -
Bcl XL
75 -
P65
25
BCL2
pcna
25-
βact

Figure 5L

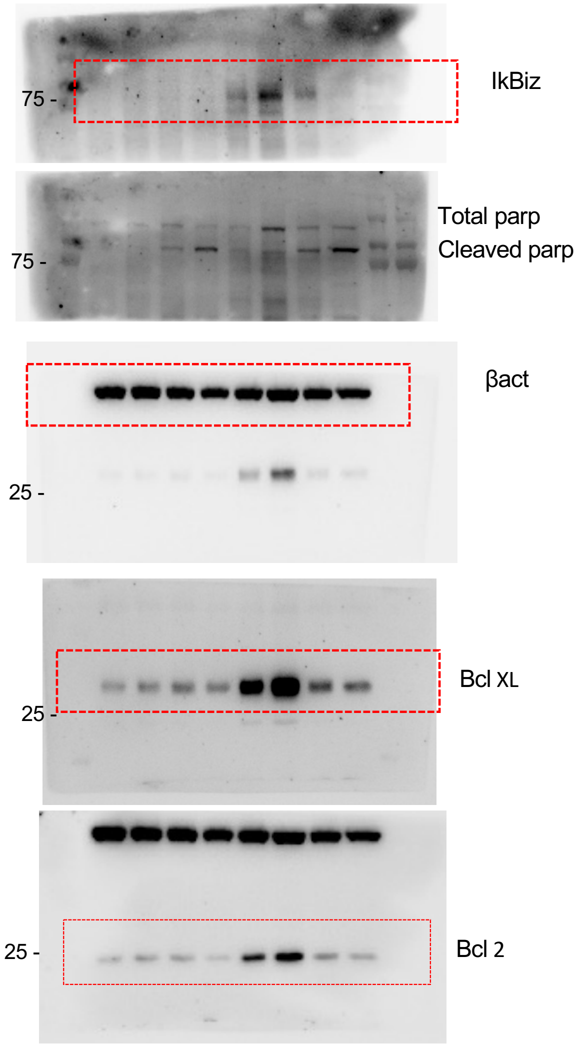

Supplementary figure Figure 3B

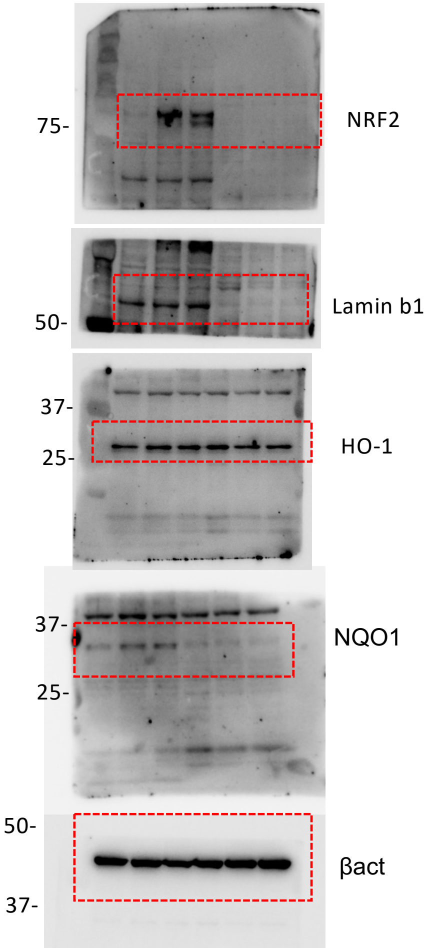

Supplementary figure Figure 3C

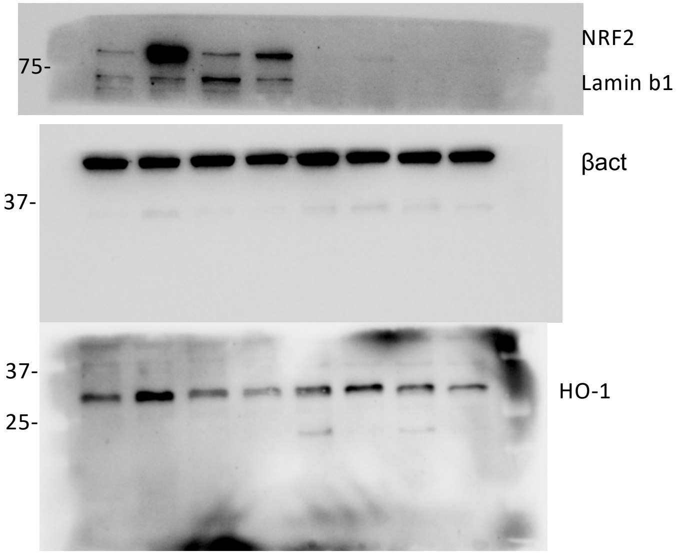

Supplement: Supplementary file 2 — Original data file [file 41419_2024_6602_MOESM2_ESM.pdf]
